# The entanglement of extracellular matrix molecules and immune checkpoint inhibitors in cancer: a systematic review of the literature

**DOI:** 10.3389/fimmu.2023.1270981

**Published:** 2023-10-03

**Authors:** Albina Fejza, Greta Carobolante, Evelina Poletto, Lucrezia Camicia, Giorgia Schinello, Emanuele Di Siena, Giuseppe Ricci, Maurizio Mongiat, Eva Andreuzzi

**Affiliations:** ^1^ Department of Biochemistry, Faculty of Medical Sciences, UBT-Higher Education Institute, Prishtina, Kosovo; ^2^ Department of Research and Diagnosis, Division of Molecular Oncology, Centro di Riferimento Oncologico di Aviano (CRO) IRCCS, Aviano, Italy; ^3^ Obstetrics and Gynecology, Institute for Maternal and Child Health, IRCCS Burlo Garofolo, Trieste, Italy; ^4^ Department of Medicine, Surgery and Health Sciences, University of Trieste, Trieste, Italy

**Keywords:** extracellular matrix, cancer, immune checkpoint inhibitors, pediatric cancer, gynecological cancer, gastrointestinal cancer, melanoma, breast cancer

## Abstract

**Introduction:**

Immune-checkpoint inhibitors (ICIs) have emerged as a core pillar of cancer therapy as single agents or in combination regimens both in adults and children. Unfortunately, ICIs provide a long-lasting therapeutic effect in only one third of the patients. Thus, the search for predictive biomarkers of responsiveness to ICIs remains an urgent clinical need. The efficacy of ICIs treatments is strongly affected not only by the specific characteristics of cancer cells and the levels of immune checkpoint ligands, but also by other components of the tumor microenvironment, among which the extracellular matrix (ECM) is emerging as key player. With the aim to comprehensively describe the relation between ECM and ICIs’ efficacy in cancer patients, the present review systematically evaluated the current literature regarding ECM remodeling in association with immunotherapeutic approaches.

**Methods:**

This review followed the Preferred Reporting Items for Systematic Reviews and Meta-analyses (PRISMA) guidelines and was registered at the International Prospective Register of Systematic Reviews (PROSPERO, CRD42022351180). PubMed, Web of Science, and Scopus databases were comprehensively searched from inception to January 2023. Titles, abstracts and full text screening was performed to exclude non eligible articles. The risk of bias was assessed using the QUADAS-2 tool.

**Results:**

After employing relevant MeSH and key terms, we identified a total of 5070 studies. Among them, 2540 duplicates, 1521 reviews or commentaries were found and excluded. Following title and abstract screening, the full text was analyzed, and 47 studies meeting the eligibility criteria were retained. The studies included in this systematic review comprehensively recapitulate the latest observations associating changes of the ECM composition following remodeling with the traits of the tumor immune cell infiltration. The present study provides for the first time a broad view of the tight association between ECM molecules and ICIs efficacy in different tumor types, highlighting the importance of ECM-derived proteolytic products as promising liquid biopsy-based biomarkers to predict the efficacy of ICIs.

**Conclusion:**

ECM remodeling has an important impact on the immune traits of different tumor types. Increasing evidence pinpoint at ECM-derived molecules as putative biomarkers to identify the patients that would most likely benefit from ICIs treatments.

**Systematic review registration:**

https://www.crd.york.ac.uk/prospero/display_record.php?ID=CRD42022351180, identifier CRD42022351180.

## 1 Introduction

The development of immunotherapy represents a revolution in the treatment of cancer and the use of immune checkpoint inhibitors (ICIs) exerts a prominent anti-tumor activity in a broad range of tumor types. Nearly half of all patients with metastatic cancer are eligible to receive ICIs, with an increasing use of these agents seen in several (neo)adjuvant and maintenance settings (1–3). ICIs are often used in combination regimens, including those involving other classes of ICI, chemotherapy, anti-angiogenic and/or targeted therapies (4). Nonetheless, despite a portion of patients display remarkable and long-lasting disease regression in response to ICIs, two thirds of the patients do not benefit from these therapies (5). This is partially due to the occurrence of primary or acquired resistance, but also to the toxicity deriving from ICs blockade, that can be severe and even life-threatening. For these reasons, it is crucial to identify the patients that could benefit of ICIs and the search for predictive biomarkers of responsiveness to ICIs remains an active area of research and an urgent clinical need.

Immune checkpoint (IC) pathways are physiologic mechanisms aimed at attenuating T cell responses to prevent autoimmunity and maintain immune homeostasis. Tumors can viciously take advantage of the immune-inhibitory pathways to limit the extent of T cell activation and maintain immune tolerance. Indeed, ICs and their ligands are frequently upregulated in the tumor microenvironment (TME) of various cancer types, thus hindering the anti-tumor immune responses (1). Hence, with the aim to revitalize the anti-tumor immune response, ICIs have been developed as promising therapeutic agents. Most of the ICIs target the cytotoxic-T-lymphocyte-associated protein 4 (CTLA-4 or CD152), the programmed cell death 1 (PD-1 or CD279) or its ligand programmed cell death ligand 1 (PD-L1 or CD274 or B7 homolog 1) (1). Many drugs inhibiting these two checkpoint axes, i.e. ipilimumab for CTLA4, nivolumab and pembrolizumab for PD-1 and atezolizumab, avelumab, and durvalumab for PD-L1, have shown clinical activity and are currently used in the clinical practice (4). In the last few years, other checkpoint molecules have been identified and an increasing number of immunotherapies is under clinical development (e.g., blockade of LAG3, CD276, TIGIT and TIM3) (4, 6).

It is now well established that the efficacy of ICIs in cancer treatment is strongly affected not only by the specific characteristics of cancer cells and the expression levels of the immune checkpoint ligands, but also by other components of the TME. Indeed, the response to ICIs highly relies on the innate immune TME constituents, e.g. macrophages and natural killer cells (NK), on the tumor hypoxic levels, as well as on the efficiency of tumor-associated vasculature (7). A key cell type that strongly shapes the TME are cancer associate fibroblasts (CAFs), that represent the most abundant stromal cells within the tumor. CAFs exert multiple functions as modulating tumor angiogenesis and metabolism, secreting growth factors and immunomodulatory cytokines and driving the remodeling of the extracellular matrix (ECM). The tumor-associated ECM displays peculiar features, such as an altered composition and stiffness, and it has been shown to educate all the cells of the TME leading to the establishment of a pro-tumoral environment. Importantly, as detailed in the present systematic review, emerging evidence are pointing at the ECM as key constituent of the TME actively modulating the efficacy of ICIs.

The ECM is a complex network of molecules which, thanks to its mechanical as well as biochemical properties, strongly impacts on all the cellular TME components, thus affecting tissue homeostasis (8, 9). The ECM is composed of fibrillar proteins (such as collagens, laminins, fibronectin, elastin), proteoglycans and several glycoproteins. For their structural features, the ECM components can interact with a variety of proteins, receptors and soluble factors, influencing the behavior of tumor cells, as well as other tumor-associated cell types such as infiltrating immune cells, stromal cells, blood vascular and lymphatic endothelial cells and pericytes (10–15). As a consequence, the ECM profoundly influences important processes driving tumor growth and progression, such as epithelial-mesenchymal transition (EMT), immune response, angiogenesis and lymphangiogenesis (16–19).

Contrary to what previously thought, the ECM is not a mere static TME component, rather it undergoes continuous dynamic remodeling as well (20, 21). ECM remodeling is mainly due to three mechanisms: 1) altered expression of the components, as reported for collagens and Tenascin-C (22); 2) increased activity of lysis oxidase (LOX) enzymes, which leads to the formation of intermolecular cross-links between collagen I fibers themselves as well as with other molecules such as collagen III and IV and fibronectin (FN), thus resulting in increased tissue stiffness; 3) high protein degradation due to the activation of proteases, among which metalloproteases (MMPs) and ADAMs are the major players (23). These processes are exacerbated in the TME, leading to the formation of an abnormal ECM which utterly differs for composition and rigidity from the healthy tissues (8). Interestingly, a mounting amount of evidence indicate that the extent of ECM remodeling and its mechanical features strongly impact on the tumor immune response (24–26).

In the light of these findings, some ECM molecules, as well as fragments deriving from their proteolytic remodeling, are emerging as putative biomarkers to delineate the immune traits of the tumors, as well as the efficacy of immunotherapies. The aim of this systematic review is to identify and summarize all the published human research studies in this context. In particular, we aim to address the following questions: Can ECM remodeling regulate the tumor immune response? Is the ECM composition impacting on the efficacy of immune checkpoint inhibitors? Can ECM molecules/fragments represent a valuable biomarker to predict the outcome of cancer patients treated with immune checkpoint inhibitors?

## 2 Methods

### 2.1 Protocol and registration

The systematic review was designed based on the Preferred Reporting Ideas for Systematic Review and Meta-analyses [PRISMA (27)] systematic review checklist and was registered on PROSPERO, (ID: CRD42022351180, review protocol link: https://www.crd.york.ac.uk/PROSPERO/display_record.php?RecordID=351180).

### 2.2 Search strategy—eligibility criteria, information sources and search terms

Original research articles written in English and published before 20 January 2023 were eligible for inclusion. We included studies reporting any relation between ECM molecules and the immune traits of the tumors, as well as the response to immune checkpoint inhibition. We included studies regarding patients diagnosed with cancer, regardless of the cancer type, disease staging and PD-L1 expression status. Our inclusion criteria did not involve any age restrictions, since we wished to comprise both young and older patients. Articles not published in English, whose full text was not available, letters to the editor, case reports, and poster presentations were excluded.

To ensure a comprehensive retrieval of all the studies relative to ECM and ICIs efficacy, we chose to exploit three relevant and reliable databases: PubMed (MEDLINE), Scopus and Web of Science. The combination of mesh terms searched in the databases were “extracellular matrix molecules” or “extracellular matrix remodeling” and “immune checkpoint inhibitors” or “immunotherapy”. Searches have also been performed using the names of the specific immune checkpoint inhibitors (Nivolumab, Pembrolizumab, Atezolizumab, Avelamab, Durvalumab, Ipilimumab, and Tremelimumab) and some specific ECM molecules (i.e. fibronectin, collagen, Emilin, tenascin-C, proteoglycans).

### 2.3 Study selection and data extraction

Duplicate articles were removed from the results of the literature search. Two independent authors screened the titles and abstracts of the remaining articles to ensure that the eligibility criteria were met. Any discrepancies between the authors were identified and discussed (with inputs from a third author if required). The remaining included articles assessed by full-text screening by two independent authors, using the same eligibility criteria.

### 2.4 Critical appraisal

Study quality and risk of bias were assessed by using the QUADAS-2 tool. The risk of bias in the studies was categorized based on the “yes” scores in the QUADAS-2 checklist. In particular, papers with all “yes” or maximum one “unclear or no” responses were classified as low risk. Instead, if two or more responses on the checklist were “unclear” or “no”, papers were attributed as unclear or high risk, respectively. If two or more responses were “unclear” and at least one response as “no” the paper was attributed as high risk. Finally, we considered the last question of the QUADAS-2 checklist (“Were all patients included in the analysis?”) as an important key factor for the evaluation of the study quality, therefore papers in which the response was “no” were classified as high risk papers.

## 3 Results

### 3.1 Literature selection

**Figure 1:**
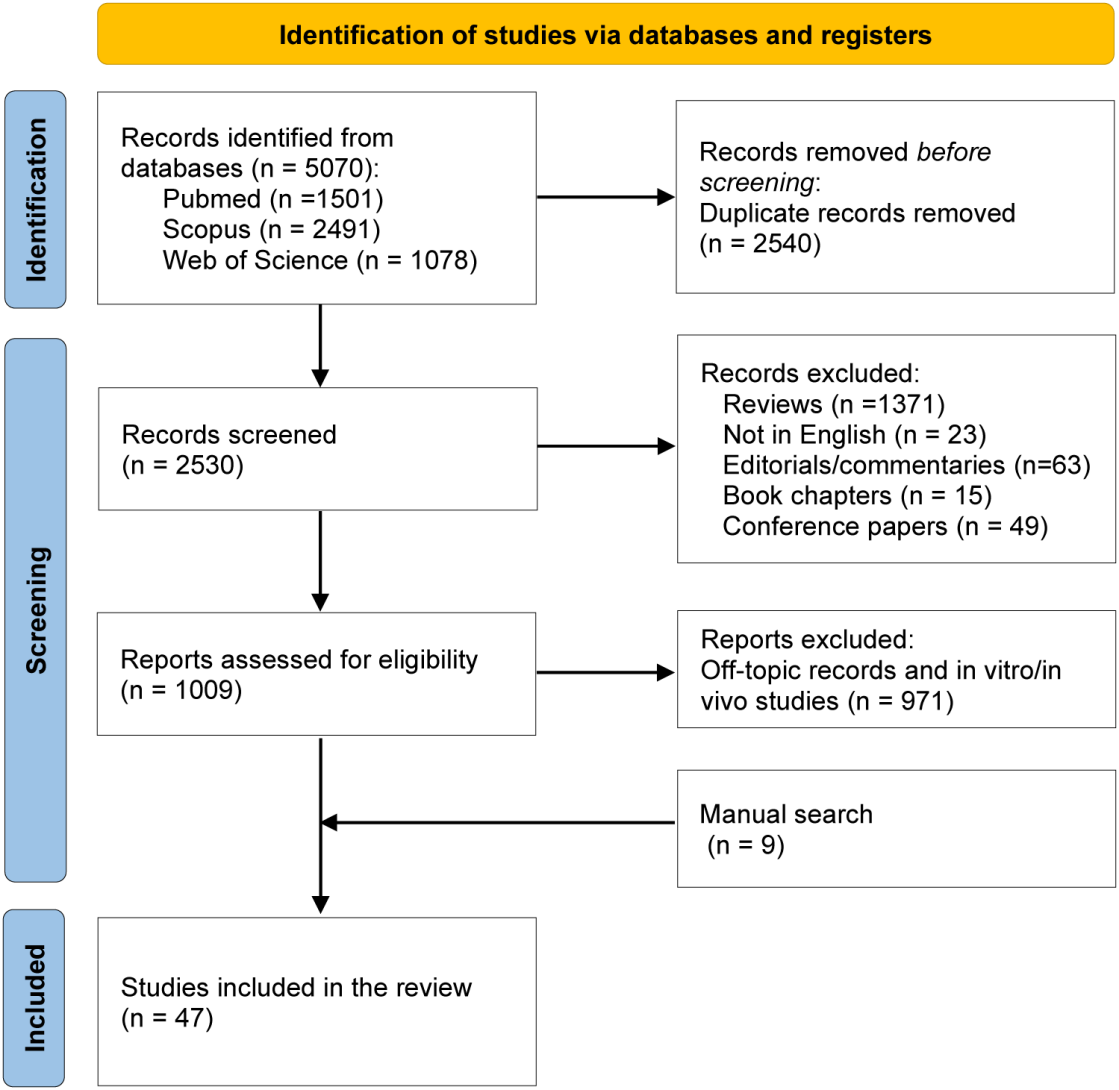
PRISMA flow diagram of the studies’ screening and selection.

The systematic search identified a total of 5,070 articles: 1,501 articles were available in PubMed, 2,491 studies in Scopus, and 1,078 in Web of Science. Among those, 2540 were duplicates and 1521 articles were excluded since the publication type did not meet the eligibility criteria (reviews, non-English articles, editorials/commentaries, book chapters, conference abstracts). Two independent authors screened a total of 1,009 articles for their relevance in the topic by assessing the title, abstract or full-text. Among the 1,009 articles, 971 studies were excluded since unrelated to ECMs, relative to immunotherapy employed to treat other diseases, and *in vitro*/*in vivo* only studies. During the screening process, nine articles not identified by the database searches but relevant for the present review were added manually to the list. As a result, 47 articles were included and analyzed in this systematic review (**Figure 1**). These studies provide a clear overview of the importance of the tumor associated ECM in determining an immunosuppressive environment within the lesions. Moreover, they highlight the association between increased ECM stiffness and remodeling processes with the response to ICIs in different tumor types, further strengthening the value of ECM-derived molecules as predictive biomarkers. A summary of the main findings of each of the 47 retained studies is provided in the following sections and in **Tables 1**, **2**.

**Table 1:** Characteristic and main findings of the included papers that show an association between ECM remodeling and tumor immune traits.

Ref	Molecule	Tumor type	Year	Enrolled Patient	Queried Databases	Association with immune traits
(28)	ABI3BP	LUAD	2023	/	TIMER, GEPIA, TCGA, HPA (n=504)	correlation with B and CD4^+^ T memory cells, Tregs, B cells, T cells, CD4^+^ T, DC activation, and Ecs
(29)	ADAM12	CRC	2021	/	Oncomine, UALCAN, TCGA, GEPIA, TIMER, TISIDB (n=86733)	correlation with CD4^+^ T, B, CD8^+^ T cells, neutrophils, macrophages, DC
(30)	ADAMs	PAAD	2020	/	TCGA (n=18313)	positive correlation with DC, B cells, neutrophils, CD8^+^ T cells, macrophages
(31)	BGN	TNBC	2022	/	TCGA, GEO (n=116)	negative correlation with CD8 ^+^ T cells
(32)		GC	2022	/	TCGA, GTEx (n=407)	positive correlation with NK cells and macrophages; negative correlation with Th17 cells
(33)	COL10A1	PAAD	2022	/	TCGA, GEO, GEPIA (n=182)	positive association with CD8^+^ T cells, M1 and M2 Mac; positive correlation with PD-L1, CTLA-4, CD73, HLA-E
(34)	COL5A1	pan-cancer	2022	/	Oncomine, TCGA, CCLE, HPA, DNMIVD,cBioPortal	association with naive B cells, memory B cells, monocytes, macrophages, CD8^+^ and CD4^+^ T cells
(35)	COL6A1	RCC	2020	161	/	association with PD-L1 expression
(36)	COL6A3	PDAC	2020	/	TCGA, GEO (n=30)	association with CD4^+^ and CD8^+^ T, B cells, neutrophils, Mac and CD; association with CTLA-4, PD-1, PD-L1, PD-L2
(37)	Collagen alignment	BC	2021	/	TCGA, GEO	negative correlation with anti-tumor T cells
(38)	Collagen I	BC	2022	30	TCGA, GEO (n=1161)	positive association with Th1 and Tregs, negative association with Th1
(39)	Collagen I, III, V	NSCLC	2021	120	/	negative association of Coll I and III with Tregs and of Coll V with NK
(40)	Collagen, Elastin	BCC	2022	22	/	association with TILs counts
(41)	CTHRC1	CRC	2022	/	TCGA, GEO (n=242)	correlation with TAMs, M2 macrophages, Tregs, T cell exhaustion, and MDSCs
(42)		GC	2022	/	TCGA, GEO, GSA (n=375)	correlation with M2 Mac, NK cells, Th1 cells and DC
(43)	EMILIN2	CRC	2022	23	TCGA (n=844)	negative association with M2 Mac; positive association with M1 Mac
(44)		LLG	2021	97	CGGA, TCGA (n=1018)	positive correlation with B cells, CD8^+^ T and CD4^+^ T cells, DC, Mac and neutrophils
(45)		CCC	2022	/	TCGA, UCSC Xena (n=531)	positive correlation with CTLA-2, PD-1, LAG3, and TIGIT
(46)	LOXL3	HCC	2021	/	TCGA, TIMER, GTEx (n=52)	positive correlation with CD4^+^ T and CD8^+^ T cells, B cells, DC, neutrophils, Mac, B cells
(47)	LOXL4	HCC	2021	90	/	association with PD-L1 expression
(48)	MMP1	HCC	2022	/	TCGA, TIMER, GEO (n=11104)	association with activated DC, Mac, CD4^+^ T cells and MDSC
(49)	MMP9	pan-cancer	2022	/	TCGA, GTEx (n=33)	positively correlates with T cells, macrophages, Th1 cells, and T cell exhaustion
(50)	PCOLCE	BC	2021	/	METABRIC (n=273)	association with PD-1/PD-L1 expression level
(51)		pan-cancer	2022	/	TCGA, CPTAC, GEO (n=33)	positive correlation with CD4^+^ and CD8^+^ memory cells, CD4^+^ T, CD8^+^ T, NK cells
(52)	PLOD2	pan-cancer	2022	/	GTEx, CCLE (n=21)	negative correlation with memory B cells, activated NK cells, CD8^+^ T cells,Treg; positive correlation with Mac
(53)	SPP1	LUAD	2021	/	TCGA, CPTAC (n=551)	correlation with low CD8^+^ Tcell infiltration and high M2-type macrophages
(54)	TNC	BC	2020	160	/	negative correlation with LC3B and CD8^+^ T cells
(55)		BC	2021	219	GEO	negative association with macrophages and CD8^+^ T cells
(56)		OSCC	2020	68	GEO	positive correlation with CD11^+^ cells and Tregs
(57)		LGG	2022	62	/	positive association with MDSC; negative association with effector T cells
(58)	Versican	NSCLC	2022	/	GEO	positive association with DC, negative correlation with CD8^+^ T cells
(59)		MPM	2022	/	TGCA (n=12)	association with PD-1 overexpression and downregulation of CD127
(60)		CCa	2010	149	/	negative association with CD8^+^ T cells
(61)		MM	2016	19	/	negative association with CD8^+^ T cells
(62)		CRC	2017	122	/	negative association with CD8^+^ T cells

BC, breast cancer; MM, myeloma; CCa, cervical cancer; MPM, pleural mesothelioma; CCC, clear cell carcinoma; CRC, colorectal cancer; NSCLC, non-small cell lung cancer; LGG, low grade glioma; OSCC, oral squamous cell carcinoma; LUAD, lung adenocarcinoma; HCC, hepatocellular carcinoma; PDAC, pancreatic ductal adenocarcinoma; PAAD, pancreatic adenocarcinoma.

**Table 2:** Characteristic and main findings of the included papers that show an association between ECM remodeling and ICIs efficacy.

Ref	Molecule	Tumor type	Year	Enrolled patients	Queried Databases	IC target	Sample type	Method	Main findings
(63)	BGN	CRC	2022	144	GEO, TCGA (N=435)	/	biopsy	RNA seq, IHC	positive association with M2 macrophages and Tregs; association with the prediction of the response to ICIs
(64)	COL6A1	BLCa	2023	58	TCGA (n=414)	PD-1	biopsy	RNA seq, IHC	predictive of poor response to anti-PD-1 treatment
(65)	Collagen fragments (C4G, PRO-C3)	CM	2020	54	/	CTLA-4	serum	ELISA	High C4G combined with low PRO-C3 predict improved OS
(66)	Collagen fragments (PRO-C3, C1M, C3M, C4M, VICM)	CM	2018	67	/	CTLA-4	serum	ELISA	High PRO-C3 and C4M independently predictive of worse OS ad PFS; high C3M/PRO-C3 and VICM independently associate with longer OS
(67)	Collagen fragments (PRO-C3, PC3X, C3M, C4M, VICM)	CM	2020	107	/	PD-1	serum	ELISA	High PRO-C3 and PC3X independently predictive of worse OS ad PFS; high C3M/PRO-C3 and VICM independently associate with improved OS
(68)	Collagen I, III	LUAD, LUSC	2020	451	TCGA (n=1580)	PD-1	biopsy	RNAseq, IHC	negative association with CD8^+^ T cells; predictive of poor survival and response to anti-PD-1
(69)	CTHRC1	GBM, LGG	2021	/	CGGA, TCGA, GDC (n=1711)	PD-1	biopsy	RNA seq	predictive value for anti-PD-1 therapy efficacy
(70)	EMILIN2	CM	2021	/	TCGA (n=477)	PD-L1	biopsy	RNA seq	negative association with the response to anti PD-L1 therapy
(71)	HAPLN3	CM	2021	/	TCGA, GEO, dbGap (n=727)	CTLA-4	biopsy	RNA seq	part of TIR signature predictive of response to anti-CTLA-4 and patients’ survival
(72)	MMP12	HCC	2021	8	TCGA, GEO (n=467)	/	biopsy	RNA seq, WB, PCR	positive correlation with CTLA-4 and PD-L1; negative association with predicted ICIs efficacy
(73)	MMP2, COL1A2	CM	2021	30	/	CTLA-4	biopsy	transcriptomic Nanostring analysis	positive association with longer OS and RFS for patients treated with anti-CTLA-4
(74)	MMP9, LOX	GBM	2023	27	TCGA, CGGA, GEO (n=1876)	PD-1, PD-L1	biopsy	RNA seq, IHC, qPCR	part of a high risk signature correlated with poor prognosis and higher response to anti-PD1/L1 therapy

BC, breast cancer; CM, cutaneous melanoma; CCa, cervical cancer; BLCa, bladder cancer; GBM, glioblastoma; LGG, low-grade glioma; CRC, colorectal cancer; LUAD, lung adenocarcinoma; LUSC, lung squamous cell carcinoma; HCC, hepatocellular carcinoma; OS, overall survival; PFS, progression-free survival.

**Figure 2:**
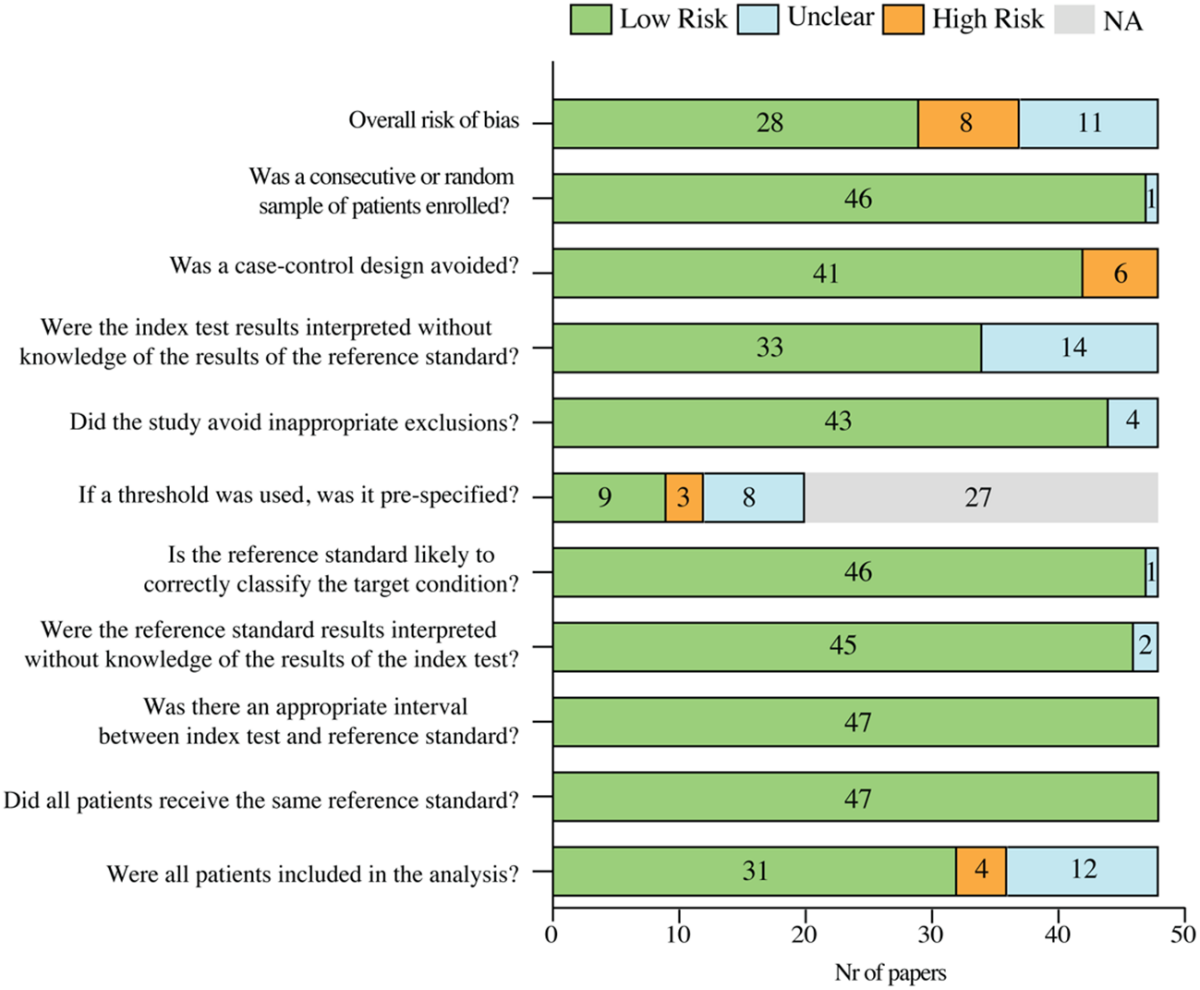
Risk of bias summary of the included papers, based on QUADAS-2 tool. NA, not applicable.

The qualitative analysis of the selected papers indicated that three points were mainly exposed to considerable bias: the design of case-control studies, the definition of a threshold and the inclusion of all the patients in the analysis. Anyway, the overall risk of bias of the included studies assessed by QUADAS-2 was low for 62% of the papers, unclear for 23% and high for 17% (**Figure 2**).

The literature search spanned from inception to 2023, however most of the papers included in the present systematic review dated from the last five years. All tumor types in adult as well as in pediatric patients were included in the search, however none of these papers dealt with pediatric cancer. Among the tumor types, the majority of the papers were related to melanoma (7/47, 14.9%), breast cancer (BC) (6/47, 12.8%), colorectal cancer (CRC) (5/47, 10.6%) and hepatocellular carcinoma (HCC) (4/47, 8.5%). Half of the identified studies were carried out exploiting the patients’ cohorts available to the research teams, whereas the other half was exclusively based on bioinformatic analyses.

### 3.2 ECM remodeling as a driver of the tumor immune environment

The ECM undergoes radical remodeling during tumor growth and progression, thereby replacing normal ECM with tumor-associated ECM (9, 75, 76). Several studies report a significant association between the altered ECM composition and the patient outcome in various cancer types, however the mechanisms underlying these changes remain elusive (10, 14, 23, 77–82). Most of these studies are focused on the effect of ECM alterations in modulating cancer cell behavior, whereas less attention has been given to their possible immunomodulatory roles. However, as described in the following paragraphs, the prominent role of some ECM components in affecting the immune cell infiltration and activity has been recently well documented.

#### 3.2.1 Altered expression of ECM components associating with the immune cell infiltration and IC expression

##### 3.2.1.1 Collagens

Among the ECM components that have been associated with the traits of the tumor immune microenvironment, collagens are the most represented (**Tables 1**, **2**).

The association between collagen deposition and the infiltration of immune cells has been well described in different cancer types. In triple negative breast cancer, high Th1 infiltration has been related to low collagen I content, whereas high Th2 and regulatory T cells (Treg) infiltration has been observed in collagen-rich lesions (38). Similar results were obtained by Yaegashi et al. in non-small cell lung carcinoma (NSCLC) (39). Yaegashi and colleagues identified three types of ECM barriers in NSCLC: the first represented by a low deposition of collagen V, the second showing an increase of collagen III and collagen I, and the third characterized by a high amount of collagen I fibers perpendicularly aligned to the tumor border. The diverse barriers were shown to be differentially permissive to immune cell infiltrates, with high density collagen V negatively correlating with NK infiltration and collagen I and III associating with decreased Treg infiltration (Yaegashi et al., 2021). A broad bioinformatic analysis showed that the collagen V gene (COL5A1) was overexpressed in a variety of tumor types including lung, breast, colorectal and gastric cancers, melanoma, liver hepatocellular carcinoma and prostate adenocarcinoma (34). The authors evidenced that COL5A1 expression increases during tumor progression, and it correlates with poor patient’s outcome in some types of cancer. Importantly, COL5A1 levels significantly correlated with the presence of a plethora of different B and T cell subpopulations. Interestingly, heterogeneity was observed among the different cancer types, allowing to conclude that the effect of COL5A1 expression is strongly dependent on the specific TME.

In other studies, some interesting associations between ECM molecules and immune checkpoints molecules have been highlighted. This is the case for renal cell carcinoma, in which the presence of COL6A1 perfectly correlates with PD-1 staining (35). Comparable results have been obtained in PDAC, in which COL6A3, SPARC and fibrillin1 (FBN1) have been correlated not only with the presence of six different immune cell types (CD4^+^ T cells, CD8^+^ T cells, B cells, neutrophils, macrophages, and dendritic cells), but also with the expression level of the checkpoint molecules CTL4, PD-1, PD-L1 and PD-L2 (36). Similarly, in pancreatic adenocarcinoma (PAAD) the presence of CD8^+^ T cells, M1 and M2 macrophages, Tregs and dendritic cells has been associated with COL10A1 expression, which seems to exert an immunosuppressive function within the TME (33). Indeed, COL10A1 also positively correlated with PD-L1 and CTLA-4, as well as with the newly identified immune checkpoints CD73 (83) and the human leukocyte antigen (HLA)-E (84). Possibly due to its involvement in the immune escape, COL10A1 associates with a poor PAAD patient prognosis (33).

Overall, this evidence has the potential to open the road towards the development of new predictive markers and novel strategies for targeted immunotherapy. In line with this hypothesis, the increased expression of COL1A2, together with other ECM-related molecules such as metalloprotease-2 (MMP2) and procollagen C-endopeptidase enhancer (PCOLCE), were shown to correlate with the survival of patients with advanced melanoma treated with neoadjuvant immunotherapy combining high-dose interferon α-2b with the anti-CTLA-4 antibody ipilimumab (73). Similarly, COL6A1 expression has been indicated as a prognostic risk gene in bladder cancer, where high COL6A1 levels being predictive of a poor response to the PD-1 inhibitor tislelizumab (64).

An important association between collagen deposition and the efficacy of ICIs has been highlighted also in lung cancer. Taking advantage of a preclinical mouse model, Peng and colleagues (68) demonstrated that collagen induces CD8^+^ T cell exhaustion through the binding with the leukocyte-associated immunoglobulin-like receptor 1 (LAIR1) acting as an immune checkpoint molecule (85). Notably, the inhibition of LOXL2 activity, which leads to the blockage of collagen deposition, sensitizes the lung tumors to anti-PD-L1 therapy. Consistently, in lung cancer patients, higher collagen I and III deposition associates with decreased CD8^+^ T cells as well as increased levels of the exhaustion markers LAIR1 and TIM-3. Of note, collagen expression was shown to predict the response to anti-PD-1 therapy and the overall survival of these patients (68).

##### 3.2.1.2 Versican

The ECM proteoglycan Versican (VCAN) exerts multiple functions by interacting with other ECM components and cell types impacting on tissue development, wound healing and cancer. Increased VCAN expression has been shown in solid tumors including ovarian, pancreatic, breast, lung, esophageal, bladder and colorectal cancer and to associate with patient’s prognosis (86, 87). Many *in vitro* and *in vivo* studies have highlighted the role of VCAN in the modulation of inflammation. Moreover, some investigations have recently shown its association with the tumor immune environment in different cancer types (88). In 2022, Yang and colleagues reported that, in pleural mesothelioma, the expression of VCAN, in association with the other ECM molecules collagen I, fibulin and NG2, identifies patients characterized by immunosuppression and resistance to chemotherapy (59). In accordance with these evidences, the presence of VCAN and the rate of its proteolytic cleavage by the specific ADAMTS1 in lung cancer has been shown to play a pivotal role in dendritic cell activation (58). In detail, VCAN is located in the peritumoral stroma of NSCLC, where the VCAN-derived proteolytic fragment versikine induces dendritic cell (DC) accumulation and activation. This, in turn, allows the interaction of DC with transiting effector CD8^+^ T cells, inducing their activation and infiltration within the tumor nest. Therefore, an active VCAN proteolysis and low total VCAN in the stroma associates with CD8^+^ T cell infiltration in NSCLC (58), myeloma (MM) (61), CRC (62) and in cervical cancer (CCa) (60). These data suggest that VCAN remodeling may be exploited as a novel immune biomarker as well as a therapeutic target to promote antitumor CD8^+^ T cell responses.

##### 3.2.1.3 Tenascin-C

The third most represented molecule in the papers analyzed in this review is tenascin-C (TNC), a highly expressed glycoprotein in malignant solid tumors, including breast cancer and oral squamous cell carcinoma (OSCC) (9, 89, 90). The functions of TNC in modulating cancer cell migration, proliferation, invasion and angiogenesis have been extensively described (91–93), however only in recent years TNC has been associated with the immune response. Analyses of TNC deposition in breast cancer, low grade glioma (LGG) and OSCC indicated that a TNC-rich stroma associates with leukocyte infiltration in the tumor nest (55–57). Murdamoothoo and colleagues demonstrated that TNC can retain T cells within the stroma by inducing and directly binding CXCL2, an important T cell chemoattractant, thus preventing their infiltration and cytotoxic activity in the tumor nest (55). A similar function was observed in OSCC, in which, through the induction of CCL21, TNC has the capability to promote the retainment of CD11c^+^ myeloid cells in the stroma leading to a more immune-suppressive environment within the tumor nest (56). In accordance with this evidence, Li and colleagues showed that, in triple-negative breast cancer (TNBC), TNC inversely correlates with CD8^+^ T-cell tumor infiltration and positively correlates with poor patient prognosis (54). Furthermore, they assessed that the expression of TNC associates with the occurrence of autophagic defects in TNBC cells, defects known to counteract T cell-mediated tumor killing. The authors demonstrated that TNC blockage can sensitize TNBC cells to the cytotoxic effect of T lymphocytes, indicating that TNC may be explored as a new potential target for TNBC treatment (54).

##### 3.2.1.4 Collagen triple helix repeat containing-1

Collagen triple helix repeat containing-1 (CTHRC1) is a secreted ECM protein transiently expressed during the repair process of injured arteries (94) and skin wound healing (95). In several solid tumors, CTHRC1 is upregulated and its expression has been associated with tumorigenesis and metastatic dissemination (96). In breast cancer, non-small cell lung cancer and oral cancer, CTHRC1 exerts a pro-tumorigenic effect by modulating the Wnt/β-catenin pathway (96). The association between CTHRC1 and the tumor immune environment has been described for the first time in a preclinical model of CRC, in which CTHRC1 was shown to promote liver metastasis by shaping the infiltrated macrophages towards a M2 phenotype through the direct interaction with the TGF-β receptors (97). This observation has been confirmed by Zhao et al., who evaluated CTHRC1 expression in gastric cancer (GC) through the integration of different datasets (42). Not only did the authors show that high CTHRC1 expression associates with worse patients’ prognosis, but they also found that it correlates with the abundance of subtypes of immune infiltrating cells. In detail, elevated CTHRC1 expression was significantly correlated with the infiltration of M2 macrophages, as well as other innate immune cells, such as NK, Th1 and DC cells. Further analyses allowed to determine that CTHRC1 is highly expressed by cancer-associated fibroblasts (CAFs) and it is present in the vascular tissue surrounding the gastric lesions, where it may favor macrophage infiltration though the interaction with CAFs via the GRN/TNFRSF1A and AnxA1/FPR1 pathways (42). CAFs are likely the major source of CTHRC1 also in CRC, in which CTHRC1 expression is upregulated and it takes part in a gene-based signature with prognostic value (41). Indeed, the upregulation of CTHRC1, together with that of the Placental Derived Growth Factor C (PDGFC), PDZ and LIM Domain 3 (PDLIM3), Neurotrimin (NTM), and Solute Carrier Family 16 Member 3 (SLC16A3) genes, positively correlates with M2 macrophages, regulatory T cells (Tregs), and myeloid-derived suppressor cells (MDSCs) infiltration, as well as T cell exhaustion and associates with poor CRC patient survival (41). Taken together, these two papers confirm the immunosuppressive role of CTHRC1 in gastrointestinal cancers. However, the association between CAF-derived CTHRC1 and the tumor immune microenvironment characteristics do not seem to be tumor type-specific. Indeed, the expression of CTHRC1, together with ATP Binding Cassette Subfamily C Member 3 (ABCC3), macrophage scavenger receptor 1 (MSR1), PDZ and LIM domain protein 1 (PDLIM1), TNF Receptor Superfamily Member 12A (TNFRSF12A), and Chitinase-3-Like Protein 2 (CHI3L2), has been identified as a CAF-related gene signature with prognostic and predictive value for glioma patients treated with anti PD-1 therapy (69).

##### 3.2.1.5 ABI family member 3 bind protein

ABI family member 3 binding protein (ABI3BP) is an ECM protein expressed in multiple organs, including the heart, kidney, lung, pancreas, and placenta, with low or variable expression in the spleen, liver, brain, bone, and skeletal muscle (98). ABI3BP expression has been associated with many physiological and pathological processes (99), and it is well known for its role in multiple cancer types, acting as a tumor suppressor by inhibiting cancer cell proliferation and migration and promoting cellular senescence (100–104). The role of ABI3BP in lung cancer has been investigated only recently and it has been indicated that this molecule is downregulated in the lesions compared to normal lung tissue and it gradually decreases as lung cancer progresses (28). Interestingly, in the same work, it has been demonstrated for the first time the association between ABI3BP expression and immune cell infiltration. Indeed, in lung cancer, ABI3BP expression positively correlates with B memory cells, CD4^+^ T memory cell rest, Tregs, CD8^+^ T cells, CD4^+^ T cells, and CD activation. Even if the molecular mechanisms affecting the immune response are still unknown, these data suggest that increased ABI3BP expression may impact on tumor progression also by modulating the tumor immune microenvironment. In accordance with this hypothesis, the expression of ABI3BP in lung cancer correlates with patient’s prognosis, with low expressing patients having a poorer outcome (28).

##### 3.2.1.6 EMILIN-2

Elastin microfibril interfacer 2 (EMILIN-2) belongs to the EDEN protein family (105–107) and is often downregulated in epithelial tumors, in which it exerts a tumor suppressive function through multiple mechanisms (11, 77, 108, 109). EMILIN-2 directly acts on the survival and proliferation of cancer cells and, like other members of the EDEN family, such as Multimerin-2 (12, 13, 110–112), it also influences angiogenesis (109). Increasing evidence pinpoint this molecule as an important immunomodulator in the TME. Recently, EMILIN-2 has been shown to affect macrophage polarization through the engagement of TLR-4 (43). Indeed, in colorectal cancer low EMILIN-2 protein levels were shown to correlate with a low M1/M2 macrophage ratio and, consistently, with poor patient prognosis. A similar observation has been made in melanoma, in which the levels of EMILIN-2 are reduced compared to the healthy tissue, and patients displaying low EMILIN2 expression are characterized by poor overall survival (70). Importantly, in these patients EMILIN-2 has been shown to associate with the efficacy of PD-L1 blockage (70), suggesting that the evaluation of EMILIN2 in the tumor tissue may entail a possible predictive value.

Contrasting results have been found in other tumor types, as in low grade glioma (44) and clear cell renal cell carcinoma (ccRCC) (45), where the upregulation of EMILIN-2 associated with poor prognosis. This evidence was supported by the positive correlation of EMILIN-2 with macrophage subsets, T reg and T cell exhaustion, overall indicating an immunosuppressive effect of EMILIN-2 in these cancer types (44). In line with these findings, in ccRCC EMILIN-2 was shown to positively associate with the levels of several checkpoint molecules including CTLA-2, PD-1, LAG3, and TIGIT (45).

##### 3.2.1.7 Biglycan

Biglycan (BGN) is an ECM proteoglycan with an essential role in mediating morphology, growth, differentiation and migration of epithelial cells and it is a well-known player in tumor development and progression (113–115). Several studies reported an up-regulation of BGN in a variety of solid tumors suggesting its potential diagnostic and prognostic value in ovarian, prostate, head and neck, gastric and colorectal cancer (116–118). However, the function of BGN in tumor immunity has just recently been assessed. He and colleagues were the first to investigate the association between BGN and immune cell infiltration (63). These authors showed that, in CRC samples, elevated levels of BGN were correlated with immunosuppressive traits and an unfavorable patients’ outcome. Indeed, BGN expression within CRC lesions positively corresponds to M2 macrophage and Treg infiltration. A bioinformatic model was applied to the same datasets indicating that CRC patients with high BGN expression levels were characterized by a higher expression of immune checkpoint molecules, as PD-L1, and were predicted to have a better response to ICIs. A similar immunosuppressive function of BGN has been found in GC (32) and in TNBC, in which high BGN levels have been negatively correlated with increased infiltration of CD8^+^ T cells and associate with poor prognosis (31).

##### 3.2.1.8 Osteopontin

Osteopontin (OPN), encoded by the SPP1 gene, is a non-collagenous bone matrix protein involved in the development of different organs (119). Many studies have assessed its role in the growth and metastatic dissemination of various solid tumors, such as breast and prostate cancer, squamous cell carcinoma, melanoma, osteosarcoma and glioblastoma, where OPN is often upregulated and correlates with a poor prognosis (120). *In vitro* and *in vivo* studies highlighted the role of OPN in determining the immune phenotype of the TME, since SPP1 expression directly correlated with CD8^+^ T cell activation and M2 macrophage polarization (121–123). However, thus far the putative association of OPN with the immune traits of the TME in human tumors has been investigated only in lung cancer. SPP1 expression was demonstrated to be higher in lung adenocarcinoma (LUAD) compared with normal lung tissue, potentially impacting on the resistance to ICIs (53). The same study indicated that a high SPP1 expression associates with poor patient prognosis and, consistently with the *in vivo* observations, SPP1 expression correlates negatively with CD8^+^ T cells and positively with M2 macrophage infiltration. Interestingly, the levels of SPP1 expression also positively correlated with the immune checkpoint CD276, particularly in patients displaying EGFR mutations (53).

##### 3.2.1.9 Hyaluronan and proteoglycan link protein 3

Hyaluronan and proteoglycan link protein 3 (HAPLN3) is an ECM linker protein involved in the binding of proteoglycans to hyaluronic acid (124). HAPLN3 is expressed in most of the tissues and it is essential for generating hyaluronic acid-dependent ECM. Some studies have reported that HAPLN3 is overexpressed in breast cancer and in CRC and its high expression is linked to cancer occurrence and metastasis (125, 126). Interestingly, the analysis on circulating tumor DNA indicated that the methylation of HAPLN3 is significantly increased in metastatic prostate cancer and serves as a post-treatment risk predictor (127). Recently, HAPLN3 together with SEL1L Family Member 3 (SEL1L3), Bone Marrow Stromal Cell Antigen 2 (BST2), and Interferon Induced Transmembrane Protein 1 (IFITM1) have been included in a four-gene signature named TIR, which highly associates with the activation of CD8^+^ T cells and immune cell infiltration in melanoma patients (71). When applied to a cohort of melanoma patients treated with the anti-CTLA-4 antibody ipilimumab, the TIR signature predicted the response to the therapy and the clinical outcome better than other known biomarkers as PD-L1 and IFN-γ, thus suggesting the potential use of the TIR signature as a predictive marker for those patients (71).

#### 3.2.2 Tumor-associated ECM as a physical barrier for immune cell infiltration

The ECM properties, due to post-translational modifications such as the bio-physical structure and the stiffness, not only affects the recruitment/activation of immune, but also per se profoundly shape the tumor immune microenvironment (128). The major ECM components involved in these two properties are collagens. These molecules are synthesized as pro-procollagens and undergo several post-translational modifications that alter their traits (23). Modifications include glycosylation, pro-peptide alignment, disulphide bond formation and hydroxylation. Importantly, lysine hydroxylation of the pro-collagen chains by lysyl hydroxylases (PLODs) allows for spontaneous triple helix formation within the cell and secretion into the extracellular space. Once secreted, the pro-peptides on the C- and N-terminus are cleaved by proteases (such as the procollagen C-endopeptidase enhancer PCOLCE) leading to the formation of collagen fibrils. For further collagen fibers assembly, lysyl oxidases (LOX) catalyses the cross-linking of collagens as well as elastin, thus modulating the ECM stiffness. Finally, collagen fibers interact with integrins and other cell surface receptors (such as RHAMM and DDR1) that apply forces leading to the alignment of the fibers (23).

In cancer, the alteration of this complex and multistep process leads to abnormal mechanical and physical properties of the ECM. The higher stiffness and density of tumor-associated ECM constitute a mechanical barrier which protects the tumor from immune cell infiltration and immune-mediated destruction. Overall the TME is less permissive to leukocyte invasion, favoring the establishment of a more tolerant immune environment and also impairing the efficacy of ICIs (129).

This aspect is well represented in the study from Byers et al, in which the authors measured the stromal fibrillar morphology within the ECM in basal cell carcinomas (BCC) (40). The authors evaluated collagen, elastin, and reticulin and defined the presence of “gaps” between the fibers as lacunarity. A higher lacunarity represents a more permissive environment and directly correlates with the infiltration of tumor-associated T lymphocytes (TIL), as assessed in BCC.

In the same view, Xu et al. showed that PLOD2 (Procollagen-Lysine,2-Oxoglutarate 5-Dioxygenase 2), a member of PLOD family which mediates the formation of stabilized collagen cross-links generating a stiffer ECM, is overexpressed in a variety of tumors including gastric, bladder, lung, breast, and head and neck squamous cell cancer (52). Notably, in GC, PLOD2 expression was negatively correlated with the presence of memory B cells, activated NK cells, plasma cells, CD8^+^ T cells, follicular helper T cells and Tregs; on the other hand, it was positively correlated with macrophages, activated mast cells, resting NK cells, CD4 memory activated T cells and CD4 memory resting T cells. Overall, PLOD2 was shown to be significantly associated with the tumor immune infiltration and with a poor patients’ outcome.

Another enzyme driving collagen rearrangements and recently associated with immune infiltrating cells is PCOLCE, which localizes in the TME of several cancer types. Bioinformatic analyses highlighted that PCOLCE is a prognostic predictor for PAAD, thymoma and CES (51). Even if the molecular mechanisms behind this observation are still unknown, PCOLCE expression correlates with the extent of CD4^+^ T, CD8^+^ T, NK cell infiltration. As well, Lecchi et al. developed a gene expression signature to identify high‐grade breast cancer patients with poor prognosis (50). PCOLCE is one of the genes taking part in the ECM3^+^/IFN^−^ signature, together with other ECM genes such as Secreted Protein Acidic And Cysteine Rich (SPARC), Biglycan, EGF Containing Fibulin Extracellular Matrix Protein 2 (EFEMP2) and the basal membrane component Nidogen 2 (NID2). In breast cancer, the ECM3^+^/IFN^−^ signature was associated with low tumor‐infiltrating lymphocytes, high levels of CD33^+^ cells, absence of PD‐1 expression or low expression of PD‐L1.

As mentioned before, ECM stiffness and structural organization are strongly regulated by the activity of LOX enzyme family, which includes LOX and LOX-like (LOXL) 1-4 (23). Due to their involvement in different processes, as linking bi-directionally the ECM and acting directly on the activation of signaling pathways regulating cancer cell survival, proliferation and differentiation, LOXs have been identified as pivotal factors in the formation and progression of different tumor types as glioma, gastric and endometrial carcinoma (130–133). Among the LOX family of enzymes, LOXL3 has also been shown to play immunomodulatory functions in the TME. A detailed bioinformatic analysis highlighted that LOXL3 is upregulated in HCC compared with normal tissues and correlates with poor prognosis (46). In the same study, for the first time LOXL3 expression has been positively correlated with the infiltration extent of multiple immune cells, among which CD8^+^ and CD4^+^ T cells and macrophages, as well as with the expression of immune checkpoint molecules such as PD-L1 and CTLA-4. A functional enrichment analysis demonstrated that this effect was mainly based on ECM organization and regulation of cell−cell adhesion (46). However, in some cases, the immunomodulatory effect of the collagen modifying enzymes is not only related to ECM remodeling but also to different mechanisms that act in a synergic fashion. As an example, the lysyl oxidase 4 (LOXL4), whose upregulation induces higher ECM stiffness, during hepatocarcinogenesis was shown to be overexpressed by macrophages and to induce an autocrine expression of PD-L1, thus contributing to maintain T‐cell exhaustion and supporting tumor progression (47). In accordance with this dual role of LOXL4 in HCC, a high expression of LOXL4 by macrophages and a low expression of the CD8^+^ T cell marker CD8A can cooperatively predict poor survival of cancer patients.

Importantly, not only the density and stiffness of the collagen matrix, but also the fiber alignment represents a barrier for immune cell infiltration. This aspect has been described in breast cancer by Sun et al. Their study reported for the first time the implication of discoidin domain receptor 1 (DDR1), a tyrosine kinase collagen receptor, in shaping the immune infiltrate of breast cancer (37). DDR1 induces immune cell exclusion through its extracellular domain by promoting the alignment of collagen fibers. In agreement with this hypothesis, in TNBC, the expression of DDR1 negatively correlates with the intratumoral abundance of anti-tumor T cells (37).

#### 3.2.3 ECM fragments as a reservoir of novel biomarkers for ICIs efficacy

ECM remodeling occurs on one side through the altered expression of the molecules, on the other side through their degradation mediated by the activation of target-specific proteases such as MMPs, disintegrins and ADAMs (23). Cancer cells and tumor associated cells express higher levels of proteases which contribute to the establishment of a pro-tumorigenic environment by multiple mechanisms (9, 23, 134). The proteolytic degradation of the ECM components allows the replacement of the normal ECM with tumor-derived ECM. This process favors the migration of cancer cells through the interstitial matrix by unlocking migratory tracks. Simultaneously, the enzymatic activity of MMPs and ADAMs induces the release of ECM-bond growth factors and proteolytic fragments, some of which exert a new biological activity respect to the molecule of origin. Some of these fragments are released in the blood stream and may be exploited to develop a liquid biopsy-based biomarkers. The association of proteolytic enzymes and ECM-derived fragments with the immune TME are described in the following paragraphs.

##### 3.2.3.1 Proteolytic enzymes

MMP-9, together with MMP-2, are the most common progression markers correlated to cancer invasion and metastasis and, recently, MMP-9 levels have been associated with the presence of immune cell infiltration, particularly with M1 and M2 macrophages, in 33 tumor types (49). In accordance, Yu and colleagues included MMP-9, together with LOX and TIMP1 in a gene-based signature, which significantly correlates with the response to anti-PD1 and anti-PD-L1 immunotherapy and overall survival of glioma patients (74). Despite contradictory results that needed further analysis, the cancer immunomodulatory function of other MMPs has also been investigated. Such is the case for MMP-1, which is known to have a role in cancer invasion and epithelial-mesenchymal transition in HCC and other tumor types (135). MMP-1 expression has been associated with the presence of anti-tumor immune cells, such as activated DC, macrophages, T helper cells and CD4^+^ T cells, as well as with the presence of MDSC cells, which, on the contrary, suppress the immune response (48). This suggests that MMP-1 functions and regulations in the TME are extremely complex and involve a number of yet elusive mechanisms. Always in the context of HCC, also MMP12 was found to be significantly increased and to associate with the CTLA-4 expression levels and with a poor ICI efficacy (72).

Like MMPs, ADAMs are often upregulated in tumors and high levels associate with a worse prognosis for the patients (136–141). Only recently, ADAMs have been linked to the immune cell infiltration and immune checkpoint molecule expression. In detail, in HCC, the expression of nine components of the ADAMs family (ADAM8,9,10,12,19,28,TS2,TS12) was shown to increase along with tumor progression and to correlate with the presence of dendritic cells, B cells, neutrophils, CD8^+^ T cells, and macrophages (30). Importantly, the same study showed that ADAM12, 19, TS2 and TS12 were positively correlated with the expression of the immune checkpoint molecules PD-1, PD-L1, PD-L2 and CTLA-4. In line with this evidence, in colorectal adenocarcinoma (COAD), one of the CRC subtypes, high ADAM12 expression associated with an altered immune cell infiltration and with a poor patients’ outcome (29). In particular, ADAM12 expression positively correlated with the presence of CD8^+^ T cells, CD4^+^ T cells, macrophages, neutrophils, and DC; on the contrary, the correlation between ADAM12 expression and presence of B cells was not significant.

##### 3.2.3.2 ECM-derived liquid biopsy biomarkers

The ECM remodeling by post-transcriptional modification enzymes and proteases generates fragments and peptides that can be detected in the peripheral blood and could be used as serological markers directly reflecting the disease and cancer progression (23, 142–144). The possibility to detect these fragments in the circulation represents an advantage compared to the analysis of tumor biopsies, considering the easy access through poorly invasive procedures, thus allowing to monitor the disease progression over time.

During collagen fibrillogenesis, the N-terminal propeptide of immature collagen is cleaved by specific proteases leading to the incorporation of the mature molecule in the ECM. The cleavage of the N-terminal region of pro-collagen III generates a fragment, named PRO-C3, which is released in the blood circulation and reflects the extent of collagen deposition, with high levels indicating an excessive collagen deposition (145). In accordance with this observation, and with the fact that collagen deposition is upregulated in immune-excluded tumors (23, 40, 129), high serum levels of PRO-C3 have been associated with poor outcome in CRC and metastatic breast cancer patients (146, 147). In melanoma, a high PRO-C3 levels correlated with low efficacy of the anti-PD-1 antibodies pembrolizumab or nivolumab (67).

The proteolytic cleavage of collagens produces the fragments C1M (collagen I), C3M (collagen III) and C4M (collagen IV) which were shown to be increased in cancer patients compared to healthy individuals (147, 148), and to associate with a poor response to anti-CTLA-4 blockage in melanoma patients (66). The same trend has been observed for PRO-C3, that together with C4M also correlated with shorter overall survival (66). In retrospective analyses, Jensen and colleagues calculated the C3M/PRO-C3 ratio as a parameter to evaluate the balance between collagen degradation and deposition, finding that a high C3M/PRO-C3 ratio was predictive of a better response to ipilimumab (66). The same observation has been observed in a prospective cohort of melanoma patients subjected to anti-PD-1 treatment, further strengthening the notion that a higher collagen degradation versus deposition favors a better outcome and response to ICIs (67).

The degradation of collagen IV by granzyme B generates a fragment distinct from C4M named C4G (149). In metastatic melanoma patients, high C4G levels at baseline corresponded to a good clinical response to anti-CTLA-4 therapy, in terms of both objective response rate and overall survival (65). Interestingly, and in line with the studies from Jensen (66) and Hurkmans (67), patients characterized by a combination of high C4G (indicating basal membrane degradation) and low PRO-C3 (suggestive of low collagen deposition) were characterized by a better chance to respond to ipilimumab compared to the patients displaying only high C4G levels (65).

Circulating fragments are generated not only by the degradation of collagen but also other ECM molecules. For example, extracellular vimentin is citrullinated and cleaved by MMPs giving rise to a fragment known as VICM (citrullinated and MMP-degraded vimentin) (150). VICM is released by tumor associated macrophages and has been detected in the serum of lung cancer patients (151, 152). In melanoma patients treated with ICIs, such as ipilimumab, nivolumab and prembolizumab, high levels of VICM before immunotherapy were linked to a survival benefit (66, 67). This finding fits well with the higher frequency of macrophages infiltrating the tumors of patients responding to ipilimumab compared with the non-responders (153).

Taken together, these studies highlight a prominent role of the ECM in affecting the immune response. From the evaluation of the 47 papers taken into account, we can infer that collagens are the most studied ECM components in this context, impacting on the infiltration and activation of immune cells by constituting a physical barrier to effector cells’ infiltration and by influencing immune cells phenotype. Moreover, collagen remodeling represents a crossing-edge process among different tumor types and provides promising valuable biomarkers for ICIs efficacy. Nonetheless, from this study we can also conclude that other ECM components as glycoproteins and proteoglycans exert a prominent role in shaping the tumor immune response despite their effect is tumor-type specific.

## 4 Discussion

**Figure 3:**
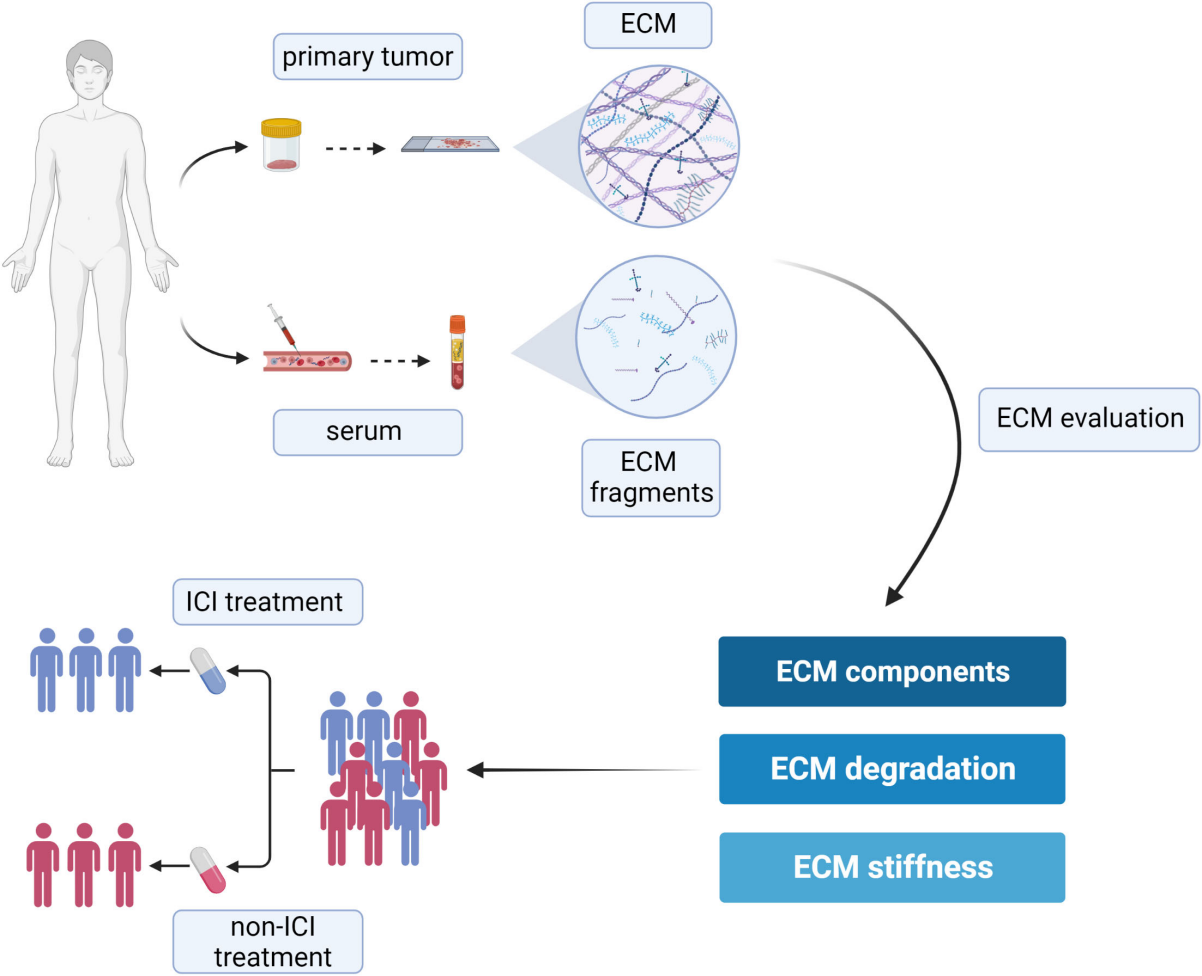
Schematic representation of the suitable approaches aimed at evaluating ECM remodeling as a tool to predict the efficacy of ICIs and to help the clinical decision-making process. Created with BioRender.com.

As a key component of the TME, the ECM is becoming a crucial source of novel diagnostic and prognostic biomarkers (75). Due to its intrinsic complexity and multimodular structure of its components, and thanks to the integration of inside-in and inside-out signals, the ECM takes part in a plethora of different processes within the tumor, being involved in a dynamic reciprocity with cancer cells, as well as tumor-associated cell types. The matrix signals affect gene expression programs shaping the phenotype of cancer cells, which in turn tightly control the ECM composition and its mechano-tensile properties. The changes in ECM composition, due to the altered expression of its components and to their overt post-transcriptional modifications, lead to the replacement of the normal ECM with a tumor-educated ECM, which supports tumor growth and progression. Only recently the abnormal ECM has also been shown to impact on the susceptibility of tumor cells to immune cell-mediated killing (154). Indeed, increasing evidence suggest that the tumor-associated ECM as well as the ECM remodeling enzymes play a vital role in the modulation of the immune response, thus impacting not only on cancer progression but also on the susceptibility to ICIs therapy. Due to the extremely complex nature of the ECM, the literature regarding this topic is intricate, spanning several matrix molecules and processes, and covering a number of different tumor types. With the aim to comprehensively describe the relation between ECM and the efficacy of ICIs in cancer patients, the present review systematically evaluated the current literature regarding this topic, highlighting the value of ECM and ECM-derived molecules as predictive biomarkers for ICIs therapy efficacy (**Figure 3**).

The literature search strategy was intended to retrieve studies dealing with both adult and pediatric patients. However, none of the papers were related to pediatric cancers, likely because, in terms of absolute numbers, pediatric cancers are relatively rare and the use of ICIs is still under evaluation for these patients (155). Also, the TME of solid pediatric tumors has not been well investigated yet, despite it is known to be characterized by low mutational burdens and by a small number of TILs compared to adult malignancies (156). In accordance with these observations, the efficacy of checkpoint inhibition is poorer compared to that observed in the adults. Unlike pediatric patients, adult patients have been treated with immunotherapy for more than a decade, with the first ICI (anti-CTLA-4) being approved for the treatment of advanced-stage melanoma in 2011. Since then, the use of ICIs as single agents or in combinatorial approaches has greatly improved tumor regression rates and long-term cancer control for melanoma patients (157). More recently, the use of ICIs in breast and colorectal cancer has been explored, however promising results have been observed only in restricted subgroups of patients (158, 159). The use of ICIs to treat these three cancer types offered the possibility to analyze numerous patients’ cohorts and to deeply investigate the characteristic of the ECM in relation to the therapy efficacy, as suggested by the fact that most of the papers included in the present systematic review regard melanoma, breast cancer and colorectal cancer.

Overall, the main processes and changes driving ECM remodeling in cancer have been well documented. However, it has become clear that each cancer type displays an unique ensemble of ECM molecules, ECM-remodeling enzymes and ECM-associated growth factors, collectively referred to as matrisome (160). This was confirmed also by the papers included in this study, with some mechanisms being strongly associated with a specific tumor type. The main ECM feature common to different tumor types is ECM stiffness, which highly impacts on immune cell infiltration representing a structural and physical barrier to the recruitment of effector T cells. An extreme matrix density and rigidity is also known to associate with impaired drug delivery to the tumors, thus pinpointing ECM stiffness as a double-edge sword deeply impaction on the efficacy of ICI (25).

On the other side, the activity of some ECM components is strongly tumor-type dependent. This can be at least partially explained by the fact that ECM molecules display multimodular structures able to simultaneously modulate various biological functions and cell types, such as CAFS, immune cells and vascular cells. Thus, the overall association between the abundance of specific ECM proteins and the tumor immune traits are the result of a tight and complex molecular crosstalk between these cell types, through mechanisms that in part still remain elusive and need further investigation. In recent years, the crosstalk between immune and endothelial cells has been investigated to assess the impact of tumor associated vascularization on ICIs efficacy. These studies highlighted the synergic beneficial effect due to the simultaneous blockage of IC and the normalization of the vascular bed, leading to the design of novel therapeutic approaches based on the combination of ICIs and angiogenic drugs. On these grounds, it would be interesting to evaluate if the levels of ECM molecules exerting a role in both immunomodulation and angiogenesis may function as valuable biomarkers to stratify and identify the patients who benefit from the combination of anti-angiogenic therapy and ICIs.

The identification of tumor-specific matrisomes suggests that tumor ECM might not only represent a valuable reservoir of predictive biomarkers but also a new therapeutic target to improve ICIs treatments. The ECM components, indeed, may be exploited as new druggable targets to act on the bio-physical properties of the matrix and, in turn to synergize with ICIs therapy. The tumor-associated ECM may be therapeutically modulated in several ways, including the targeting of single ECM molecules or ECM-remodeling enzymes. For example, the administration of recombinant hyaluronidase to reduce hyaluronan accumulation has been used in phase I and II clinical trials in combination with pembrolizumab and atezolizumab for the treatment of stomach, lung and pancreatic cancer (161, 162). These trials will open the road for the clinical evaluation of other ECM/ICIs-based combinatorial therapy, as suggested by the promising data regarding the targeting of TNC and versican, which improved T cell mediated cancer cell killing in preclinical models (54). In addition, the ECM is under evaluation as a putative mean to improve drug delivery to the tumors. The use of tumor ECM-specific antibodies fused with cytokines (i.e. IL-2 and IL-12) or compounds (i.e. sunitinib) have in fact been shown to lead to increased concentrations of the drugs within the tumors, reduced severity of the side effects, and enhanced therapy efficacy (163–167). In the future, it is conceivable that similar approaches may be exploited also for the delivery of ICIs.

The potential weakness of the present systematic review resided in the fact that many of the studies are based on bioinformatic analyses. This represents a major limit since the altered mRNA levels not always coincide with the same alterations in the protein content. And this is particularly true when dealing with ECM molecules, which are extensively regulated not only at the transcriptional, but also post-translational level and undergo continuous remodeling. Nonetheless, we chose to comprise these studies since they were based on solid and strong results and provided deeper insights in the association between ECM and immune response, building the grounds for the development of new putative markers. Studies base on proteomic databases would certainly serve better this purpose, however these databases are limited compared to the RNAseq-based datasets. On these bases, we consider that more efforts should be put to attain a comprehensive proteomic profiling of the TME.

The use of ICIs represents an important therapeutic option for cancer treatment, with subgroups of patients gaining major and long-term benefits. Nonetheless, a large number of patients showing scarce response to ICIs and some experiencing unwanted side effects. For these reasons, the identification of the patients that would better benefit from immunotherapies is key to avoid over-treatments and unnecessary side effects. In addition, this approach would allow the National Health Systems to optimize more efficiently the resources. Indeed, many investigations aimed at identifying reliable predictive biomarkers for ICIs efficacy are ongoing (168). These approaches span from the analysis of cancer cell intrinsic features, such as the presence of specific gene mutations and their metabolic status, to the characterization of tumor associated stroma cells (169–172). Indeed, CAFs represent not only a promising prognostic biomarker (173, 174), but may also grant the possibility to predict ICIs efficacy, as highlighted in the present work. In this scenario, the ECM and its remodeling are entangled with the CAFs function and represent a passepartout to unravel the traits of the tumor immune environment. Indeed, the present systematic review indicates that ECM remodeling and ECM-derived fragments can represent a widow’s cruse for the development of valuable biomarkers to predict the clinical outcome and to help identifying the patients that will better benefit from ICIs therapies. Importantly, the identification of circulating ECM fragments with predictive value would provide a fast and easily accessible liquid-biopsy based test to help clinicians to determine the most appropriate therapy for each patient. Nonetheless, further validations are needed, and it will be crucial to identify a threshold to successfully apply patient-tailored therapies. Given the complex network of ECM molecules, most of which have still not been evaluated in this context, we envision that the ECM will be extensively exploited for the development of new biomarkers to predict immunotherapy efficacy.

## Data availability statement

The datasets presented in this study can be found in online repositories. The names of the repository/repositories and accession number(s) can be found below: https://doi.org/10.5281/zenodo.8348665.

## Author contributions

AF: Data curation, Writing – original draft, Formal Analysis, Investigation, Methodology. GC: Data curation, Formal Analysis, Investigation, Writing – original draft. EP: Formal Analysis, Writing – review & editing. LC: Formal Analysis, Writing – review & editing. GS: Formal Analysis, Writing – review & editing. ED: Formal Analysis, Writing – review & editing. GR: Writing – review & editing, Funding acquisition, Project administration. MM: Funding acquisition, Supervision, Writing – original draft. EA: Conceptualization, Data curation, Writing – original draft.

## References

[1] Wei SC Duffy CR Allison JP . Fundamental mechanisms of immune checkpoint blockade therapy. Cancer Discovery (2018) 8(9):1069–86. doi: 10.1158/2159-8290.CD-18-0367 30115704

[2] Johnson DB Nebhan CA Moslehi JJ Balko JM . Immune-checkpoint inhibitors: long-term implications of toxicity. Nat Rev Clin Oncol (2022) 19(4):254–67. doi: 10.1038/s41571-022-00600-w PMC8790946 35082367

[3] Lu Y Wang W Wang F . Clinical benefits of PD-1 inhibitors in specific subgroups of patients with advanced esophageal squamous cell carcinoma: a systematic review and meta-analysis of phase 3 randomized clinical trials. Front Immunol (2023) 14:14. doi: 10.3389/fimmu.2023.1171671 PMC10185849 37205107

[4] Iranzo P Callejo A Assaf JD Molina G Lopez DE Garcia-Illescas D . Overview of checkpoint inhibitors mechanism of action: role of immune-related adverse events and their treatment on progression of underlying cancer. Front Med (2022) 9:875974. doi: 10.3389/fmed.2022.875974 PMC9189307 35707528

[5] Wang J Yang T Xu J . Therapeutic development of immune checkpoint inhibitors. Adv Exp Med Biol (2020) 1248:619–49. doi: 10.1007/978-981-15-3266-5_23 32185726

[6] Picarda E Ohaegbulam KC Zang X . Molecular pathways: targeting B7-H3 (CD276) for human cancer immunotherapy. Clin Cancer Res (2016) 22(14):3425–31. doi: 10.1158/1078-0432.CCR-15-2428 PMC4947428 27208063

[7] Petitprez F Meylan M de Reyniès A Sautès-Fridman C Fridman WH . The tumor microenvironment in the response to immune checkpoint blockade therapies. Front Immunol (2020) 11:784. doi: 10.3389/fimmu.2020.00784 32457745 PMC7221158

[8] Deng B Zhao Z Kong W Han C Shen X Zhou C . Biological role of matrix stiffness in tumor growth and treatment. J Transl Med (2022) 20(1):540. doi: 10.1186/s12967-022-03768-y 36419159 PMC9682678

[9] Fejza A Camicia L Poletto E Carobolante G Mongiat M Andreuzzi E . ECM remodeling in squamous cell carcinoma of the aerodigestive tract: pathways for cancer dissemination and emerging biomarkers. Cancers (Basel) (2021) 13(11):2759. doi: 10.3390/cancers13112759 34199373 PMC8199582

[10] Chen CG Iozzo RV . Extracellular matrix guidance of autophagy: a mechanism regulating cancer growth. Open Biol (2022) 12(1):210304. doi: 10.1098/rsob.210304 34982945 PMC8727153

[11] Andreuzzi E Fejza A Capuano A Poletto E Pivetta E Doliana R . Deregulated expression of Elastin Microfibril Interfacer 2 (EMILIN2) in gastric cancer affects tumor growth and angiogenesis. Matrix BiolPlus (2020) 7(2590-0285(2590-0285 (Linking):100029. doi: 10.1016/j.mbplus.2020.100029 PMC7852313 33543026

[12] Andreuzzi E Colladel R Pellicani R Tarticchio G Cannizzaro R Spessotto P . The angiostatic molecule Multimerin 2 is processed by MMP-9 to allow sprouting angiogenesis. Matrix Biol (2017) 1569-1802(Electronic):40–53. doi: 10.1016/j.matbio.2017.04.002 28435016

[13] Colladel R Pellicani R Andreuzzi E Paulitti A Tarticchio G Todaro F . MULTIMERIN2 binds VEGF-A primarily via the carbohydrate chains exerting an angiostatic function and impairing tumor growth. Oncotarget (2015) 7(2):2022–37. doi: 10.18632/oncotarget.6515 PMC4811514 26655500

[14] Neill T Xie C Iozzo RV . Decorin evokes reversible mitochondrial depolarization in carcinoma and vascular endothelial cells. Am J Physiol-Cell Physiol (2022) 323(5):C1355–73. doi: 10.1152/ajpcell.00325.2022 PMC9602711 36036446

[15] Faisal SM Comba A Varela ML Argento AE Brumley E Abel C . The complex interactions between the cellular and non-cellular components of the brain tumor microenvironmental landscape and their therapeutic implications. Front Oncol (2022) 12:1005069. doi: 10.3389/fonc.2022.1005069 36276147 PMC9583158

[16] Danussi C Spessotto P Petrucco A Wassermann B Sabatelli P Montesi M . Emilin1 deficiency causes structural and functional defects of lymphatic vasculature. Mol Cell Biol (2008) 28(0270-7306(0270-7306 (Linking):4026–39. doi: 10.1128/MCB.02062-07 PMC2423131 18411305

[17] Mongiat M Buraschi S Andreuzzi E Neill T Iozzo RV . Extracellular matrix: the gatekeeper of tumor angiogenesis. Biochem Soc Trans (2019) 47(5):1543–55. doi: 10.1042/BST20190653 31652436

[18] Spada S Tocci A Di Modugno F Nisticò P . Fibronectin as a multiregulatory molecule crucial in tumor matrisome: from structural and functional features to clinical practice in oncology. J Exp Clin Cancer Res (2021) 40(1):102. doi: 10.1186/s13046-021-01908-8 33731188 PMC7972229

[19] van Tienderen GS Rosmark O Lieshout R Willemse J de Weijer F Elowsson Rendin L . Extracellular matrix drives tumor organoids toward desmoplastic matrix deposition and mesenchymal transition. Acta Biomater (2023) 158:115–31. doi: 10.1016/j.actbio.2022.11.038 36427688

[20] Karamanos NK Theocharis AD Neill T Iozzo RV . Matrix modeling and remodeling: A biological interplay regulating tissue homeostasis and diseases. Matrix Biol (2019) 75–76:1–11. doi: 10.1016/j.matbio.2018.08.007 PMC6377817 30130584

[21] He Z Xin Z Yang Q Wang C Li M Rao W . Mapping the single-cell landscape of acral melanoma and analysis of the molecular regulatory network of the tumor microenvironments. Elife (2022) 11. doi: 10.7554/eLife.78616.sa2 PMC9398445 35894206

[22] Virtuoso A De Luca C Cirillo G Riva M Romano G Bentivegna A . Tumor microenvironment and immune escape in the time course of glioblastoma. Mol Neurobiol (2022) 59(11):6857–73. doi: 10.1007/s12035-022-02996-z PMC9525332 36048342

[23] Winkler J Abisoye-Ogunniyan A Metcalf KJ Werb Z . Concepts of extracellular matrix remodelling in tumour progression and metastasis. Nat Commun (2020) 11(1):5120. doi: 10.1038/s41467-020-18794-x 33037194 PMC7547708

[24] Hongu T Pein M Insua-Rodríguez J Gutjahr E Mattavelli G Meier J . Perivascular tenascin C triggers sequential activation of macrophages and endothelial cells to generate a pro-metastatic vascular niche in the lungs. Nat Cancer (2022) 3(4):486–504. doi: 10.1038/s43018-022-00353-6 35469015 PMC9046090

[25] Zhu P Lu H Wang M Chen K Chen Z Yang L . Targeted mechanical forces enhance the effects of tumor immunotherapy by regulating immune cells in the tumor microenvironment. Cancer Biol Med (2023) 20(1):44–55. doi: 10.20892/j.issn.2095-3941.2022.0491 36647779 PMC9843446

[26] Larsen AMH Kuczek DE Kalvisa A Siersbæk MS Thorseth ML Johansen AZ . Collagen density modulates the immunosuppressive functions of macrophages. J Immunol (2020) 205(5):1461–72. doi: 10.4049/jimmunol.1900789 32839214

[27] Page MJ McKenzie JE Bossuyt PM Boutron I Hoffmann TC Mulrow CD . The PRISMA 2020 statement: an updated guideline for reporting systematic reviews. BMJ (2021) 372:n71. doi: 10.1136/bmj.n71 33782057 PMC8005924

[28] Feng Y Han X Zhang Z Qiao H Tang H . ABI3BP is a prognosis biomarker related with clinicopathological features and immunity infiltration of lung tumor. Front Genet (2022) 13:1085785. doi: 10.3389/fgene.2022.1085785 36744181 PMC9894588

[29] Huang Z Lai H Liao J Cai J Li B Meng L . Upregulation of ADAM12 is associated with a poor survival and immune cell infiltration in colon adenocarcinoma. Front Oncol (2021) 16(11):729230. doi: 10.3389/fonc.2021.729230 PMC8483634 34604068

[30] Qi B Liu H Dong Y Shi X Zhou Q Zeng F . The nine ADAMs family members serve as potential biomarkers for immune infiltration in pancreatic adenocarcinoma. PeerJ (2020) 8:e9736. doi: 10.7717/peerj.9736 33062410 PMC7532768

[31] Zheng S Zou Y Tang Y Yang A Liang JY Wu L . Landscape of cancer-associated fibroblasts identifies the secreted biglycan as a protumor and immunosuppressive factor in triple-negative breast cancer. Oncoimmunology (2022) 11(1):2020984. doi: 10.1080/2162402X.2021.2020984 35003899 PMC8741292

[32] Zhang S Yang H Xiang X Liu L Huang H Tang G . BGN may be a potential prognostic biomarker and associated with immune cell enrichment of gastric cancer. Front Genet (2022) 13:765569. doi: 10.3389/fgene.2022.765569 35154268 PMC8826557

[33] Liu Q Zhao H Guo Y Zhang K Shang F Liu T . Bioinformatics-based analysis: noncoding RNA-mediated COL10A1 is associated with poor prognosis and immune cell infiltration in pancreatic cancer. J Healthc Eng (2022) ;2022:7904982. doi: 10.1155/2022/7904982 36105715 PMC9467764

[34] Zhu H Hu X Feng S Jian Z Xu X Gu L . The hypoxia-related gene COL5A1 is a prognostic and immunological biomarker for multiple human tumors. Oxid Med Cell Longev (2022) 2022:6419695. doi: 10.1155/2022/6419695 35082969 PMC8786464

[35] Karabulut YY Köse EÇ Bozlu M Tuncel F Yüksek GE Etit D . The role of COL6A1and PD-1 expressions in renal cell carcinoma. Turk J Urol (2020) 46(4):282–7. doi: 10.5152/tud.2020.20062 PMC7360155 32479254

[36] Luan H Zhang C Zhang T He Y Su Y Zhou L . Identification of key prognostic biomarker and its correlation with immune infiltrates in pancreatic ductal adenocarcinoma. Dis Markers (2020) 2020:8825997. doi: 10.1155/2020/8825997 32934754 PMC7479484

[37] Sun X Wu B Chiang HC Deng H Zhang X Xiong W . Tumour DDR1 promotes collagen fibre alignment to instigate immune exclusion. Nature (2021) 599(7886):673–8. doi: 10.1038/s41586-021-04057-2 PMC8839149 34732895

[38] Zhou Y Tian Q Gao H Zhu L Zhang Y Zhang C . Immunity and extracellular matrix characteristics of breast cancer subtypes based on identification by T helper cells profiling. Front Immunol (2022) 13:859581. doi: 10.3389/fimmu.2022.859581 35795662 PMC9251002

[39] Yaegashi LB Baldavira CM Prieto TG MaChado-Rugolo J Velosa APP da Silveira LKR . *In situ* overexpression of matricellular mechanical proteins demands functional immune signature and mitigates non-small cell lung cancer progression. Front Immunol (2021) 12:714230. doi: 10.3389/fimmu.2021.714230 34484217 PMC8415570

[40] Byers C Gill M Kurtansky NR Alessi-Fox C Harman M Cordova M . Tertiary lymphoid structures accompanied by fibrillary matrix morphology impact anti-tumor immunity in basal cell carcinomas. Front Med (Lausanne) (2022) 9:981074. doi: 10.3389/fmed.2022.981074 36388913 PMC9647637

[41] Wang J Akter R Shahriar MF Uddin MN . Cancer-associated stromal fibroblast-derived transcriptomes predict poor clinical outcomes and immunosuppression in colon cancer. Pathol Oncol Res (2022) 28:1610350. doi: 10.3389/pore.2022.1610350 35991839 PMC9385976

[42] Zhao L Wang W Niu P Luan X Zhao D Chen Y . The molecular mechanisms of CTHRC1 in gastric cancer by integrating TCGA, GEO and GSA datasets. Front Genet (2022) 13:900124. doi: 10.3389/fgene.2022.900124 35928443 PMC9343808

[43] Andreuzzi E Fejza A Polano M Poletto E Camicia L Carobolante G . Colorectal cancer development is affected by the ECM molecule EMILIN-2 hinging on macrophage polarization *via* the TLR-4/MyD88 pathway. J Exp Clin Cancer Res (2022) 41(1):60. doi: 10.1186/s13046-022-02271-y 35148799 PMC8840294

[44] Wang Lc Cui WI Zhang Z Tan Zl Lv Q Chen SH . Expression, methylation and prognostic feature of EMILIN2 in Low-Grade-Glioma. Brain Res Bulletin (2021) 175:26–36. Cui W yao. doi: 10.1016/j.brainresbull.2021.07.013 34280481

[45] Zhao G Zheng J Tang K Chen Q . EMILIN2 is associated with prognosis and immunotherapy in clear cell renal cell carcinoma. Front Genet (2022) 13:1058207. doi: 10.3389/fgene.2022.1058207 36544490 PMC9760906

[46] Wang N Zhou X Tang F Wang X Zhu X . Identification of LOXL3-associating immune infiltration landscape and prognostic value in hepatocellular carcinoma. Virchows Arch (2021) 479(6):1153–65. doi: 10.1007/s00428-021-03193-4 34448895

[47] Tan HY Wang N Zhang C Chan YT Yuen MF Feng Y . Lysyl oxidase-like 4 fosters an immunosuppressive microenvironment during hepatocarcinogenesis. Hepatology (2021) 73(6):2326–41. doi: 10.1002/hep.31600 PMC8251926 33068461

[48] Dai L Mugaanyi J Cai X Dong M Lu C Lu C . Comprehensive bioinformatic analysis of MMP1 in hepatocellular carcinoma and establishment of relevant prognostic model. Sci Rep (2022) 12:13639. doi: 10.1038/s41598-022-17954-x 35948625 PMC9365786

[49] Zeng Y Gao M Lin D Du G Cai Y . Prognostic and immunological roles of MMP-9 in pan-cancer. BioMed Res Int (2022) 2022:2592962. doi: 10.1155/2022/2592962 35178444 PMC8844435

[50] Lecchi M Verderio P Cappelletti V De Santis F Paolini B Monica M . A combination of extracellular matrix- and interferon-associated signatures identifies high-grade breast cancers with poor prognosis. Mol Oncol (2021) 15(5):1345–57. doi: 10.1002/1878-0261.12912 PMC8096783 33523584

[51] Gao H Li Q . A pan-cancer analysis of the oncogenic role of procollagen C-endopeptidase enhancer (PCOLCE) in human. Med (Baltimore) (2022) 101(52):e32444. doi: 10.1097/MD.0000000000032444 PMC9803463 36596064

[52] Xu Q Kong N Zhao Y Wu Q Wang X Xun X . Pan-cancer analyses reveal oncogenic and immunological role of PLOD2. Front Genet (2022) 13:864655. doi: 10.3389/fgene.2022.864655 35586565 PMC9108334

[53] Zheng Y Hao S Xiang C Han Y Shang Y Zhen Q . The correlation between SPP1 and immune escape of EGFR mutant lung adenocarcinoma was explored by bioinformatics analysis. Front Oncol (2021) 11:592854. doi: 10.3389/fonc.2021.592854 34178613 PMC8222997

[54] Li ZL Zhang HL Huang Y Huang JH Sun P Zhou NN . Autophagy deficiency promotes triple-negative breast cancer resistance to T cell-mediated cytotoxicity by blocking tenascin-C degradation. Nat Commun (2020) 11(1):3806. doi: 10.1038/s41467-020-17395-y 32732922 PMC7393512

[55] Murdamoothoo D Sun Z Yilmaz A Riegel G Abou-Faycal C Deligne C . Tenascin-C immobilizes infiltrating T lymphocytes through CXCL12 promoting breast cancer progression. EMBO Mol Med (2021) 13(6):e13270. doi: 10.15252/emmm.202013270 33988305 PMC8185552

[56] Spenlé C Loustau T Murdamoothoo D Erne W Beghelli-de la Forest Divonne S Veber R . Tenascin-C orchestrates an immune-suppressive tumor microenvironment in oral squamous cell carcinoma. Cancer Immunol Res (2020) 8(9):1122–38. doi: 10.1158/2326-6066.CIR-20-0074 32665262

[57] Zhang P Liu G Hu J Chen S Wang B Peng P . Tenascin-C can serve as an indicator for the immunosuppressive microenvironment of diffuse low-grade gliomas. Front Immunol (2022) 13:824586. doi: 10.3389/fimmu.2022.824586 35371015 PMC8966496

[58] Papadas A Deb G Cicala A Officer A Hope C Pagenkopf A . Stromal remodeling regulates dendritic cell abundance and activity in the tumor microenvironment. Cell Rep (2022) 40(7):111201. doi: 10.1016/j.celrep.2022.111201 35977482 PMC9402878

[59] Yang H Berezowska S Dorn P Zens P Chen P Peng RW . Tumor-infiltrating lymphocytes are functionally inactivated by CD90+ stromal cells and reactivated by combined Ibrutinib and Rapamycin in human pleural mesothelioma. Theranostics (2022) 12(1):167–85. doi: 10.7150/thno.61209 PMC8690914 34987640

[60] Gorter A Zijlmans HJ van Gent H Trimbos JB Fleuren GJ Jordanova ES . Versican expression is associated with tumor-infiltrating CD8-positive T cells and infiltration depth in cervical cancer. Mod Pathol (2010) 23(12):1605–15. doi: 10.1038/modpathol.2010.154 20729814

[61] Hope C Foulcer S Jagodinsky J Chen SX Jensen JL Patel S . Immunoregulatory roles of versican proteolysis in the myeloma microenvironment. Blood (2016) 128(5):680–5. doi: 10.1182/blood-2016-03-705780 PMC4974200 27259980

[62] Hope C Emmerich PB Papadas A Pagenkopf A Matkowskyj KA Van De Hey DR . Versican-derived matrikines regulate Batf3-dendritic cell differentiation and promote T-cell infiltration in colorectal cancer. J Immunol (2017) 199(5):1933–41. doi: 10.4049/jimmunol.1700529 PMC5568487 28754680

[63] He ZX Zhao SB Fang X JF E Fu HY Song YH . Prognostic and predictive value of BGN in colon cancer outcomes and response to immunotherapy. Front Oncol (2021) 11:761030. doi: 10.3389/fonc.2021.761030 35096572 PMC8790701

[64] Zhang X Liu J Yang X Jiao W Shen C Zhao X . High expression of COL6A1 predicts poor prognosis and response to immunotherapy in bladder cancer. Cell Cycle (2023) 22(5):610–8. doi: 10.1080/15384101.2022.2154551 PMC9928451 36474424

[65] Jensen C Sinkeviciute D Madsen DH Önnerfjord P Hansen M Schmidt H . Granzyme B degraded type IV collagen products in serum identify melanoma patients responding to immune checkpoint blockade. Cancers (Basel) (2020) 12(10):2786. doi: 10.3390/cancers12102786 32998446 PMC7601429

[66] Jensen C Madsen DH Hansen M Schmidt H Svane IM Karsdal MA . Non-invasive biomarkers derived from the extracellular matrix associate with response to immune checkpoint blockade (anti-CTLA-4) in metastatic melanoma patients. J Immunother Cancer (2018) 6(1):152. doi: 10.1186/s40425-018-0474-z 30567561 PMC6300009

[67] Hurkmans DP Jensen C Koolen SLW Aerts J Karsdal MA Mathijssen RHJ . Blood-based extracellular matrix biomarkers are correlated with clinical outcome after PD-1 inhibition in patients with metastatic melanoma. J Immunother Cancer (2020) 8(2):e001193. doi: 10.1136/jitc-2020-001193 33093157 PMC7583811

[68] Peng DH Rodriguez BL Diao L Chen L Wang J Byers LA . Collagen promotes anti-PD-1/PD-L1 resistance in cancer through LAIR1-dependent CD8+ T cell exhaustion. Nat Commun (2020) 11:4520. doi: 10.1038/s41467-020-18298-8 32908154 PMC7481212

[69] Chen Z Zhuo S He G Tang J Hao W Gao WQ . Prognosis and immunotherapy significances of a cancer-associated fibroblasts-related gene signature in gliomas. Front Cell Dev Biol (2021) 9:721897. doi: 10.3389/fcell.2021.721897 34778248 PMC8586504

[70] Fejza A Polano M Camicia L Poletto E Carobolante G Toffoli G . The efficacy of anti-PD-L1 treatment in melanoma is associated with the expression of the ECM molecule EMILIN2. Int J Mol Sci (2021) 22(14):7511. doi: 10.3390/ijms22147511 34299131 PMC8306837

[71] Mei Y Chen MJM Liang H Ma L . A four-gene signature predicts survival and anti-CTLA4 immunotherapeutic responses based on immune classification of melanoma. Commun Biol (2021) 4:383. doi: 10.1038/s42003-021-01911-x 33753855 PMC7985195

[72] Huang H Hu Y Guo L Wen Z . Integrated bioinformatics analyses of key genes involved in hepatocellular carcinoma immunosuppression. Oncol Lett (2021) 22(6):830. doi: 10.3892/ol.2021.13091 34691257 PMC8527569

[73] Khunger A Piazza E Warren S Smith TH Ren X White A . CTLA-4 blockade and interferon-α induce proinflammatory transcriptional changes in the tumor immune landscape that correlate with pathologic response in melanoma. PloS One (2021) 16(1):e0245287. doi: 10.1371/journal.pone.0245287 33428680 PMC7799833

[74] Yu H Wang M Wang X Jiang X . Immune-related matrisomes are potential biomarkers to predict the prognosis and immune microenvironment of glioma patients. FEBS Open Bio (2023) 13(2):307–22. doi: 10.1002/2211-5463.13541 PMC9900094 36560848

[75] Iozzo RV Theocharis AD Neill T Karamanos NK . Complexity of matrix phenotypes. Matrix Biol Plus (2020) 6–7:100038. doi: 10.1016/j.mbplus.2020.100038 PMC7852209 33543032

[76] Girigoswami K Saini D Girigoswami A . Extracellular matrix remodeling and development of cancer. Stem Cell Rev Rep (2021) 17(3):739–47. doi: 10.1007/s12015-020-10070-1 33128168

[77] Mongiat M Ligresti G Marastoni S Lorenzon E Doliana R Colombatti A . Regulation of the extrinsic apoptotic pathway by the extracellular matrix glycoprotein EMILIN2. Mol Cell Biol (2007) 27(0270-7306(0270-7306 (Linking):7176–87. doi: 10.1128/MCB.00696-07 PMC2168889 17698584

[78] Mongiat M Marastoni S Ligresti G Lorenzon E Schiappacassi M Perris R . The extracellular matrix glycoprotein elastin microfibril interface located protein 2: a dual role in the tumor microenvironment. Neoplasia (2010) 12(4):294–304. doi: 10.1593/neo.91930 20360940 PMC2847737

[79] Chakravarthy A Khan L Bensler NP Bose P De Carvalho DD . TGF-β-associated extracellular matrix genes link cancer-associated fibroblasts to immune evasion and immunotherapy failure. Nat Commun (2018) 9(1):4692. doi: 10.1038/s41467-018-06654-8 30410077 PMC6224529

[80] Ahluwalia P Ahluwalia M Mondal AK Sahajpal N Kota V Rojiani MV . Prognostic and therapeutic implications of extracellular matrix associated gene signature in renal clear cell carcinoma. Sci Rep (2021) 11(1):7561. doi: 10.1038/s41598-021-86888-7 33828127 PMC8026590

[81] Fromme JE Zigrino P . The role of extracellular matrix remodeling in skin tumor progression and therapeutic resistance. Front Mol Biosci (2022) 9:864302. doi: 10.3389/fmolb.2022.864302 35558554 PMC9086898

[82] Evers M Schreder M Stühmer T Jundt F Ebert R Hartmann TN . Prognostic value of extracellular matrix gene mutations and expression in multiple myeloma. Blood Cancer J (2023) 13(1):43. doi: 10.1038/s41408-023-00817-7 36959208 PMC10036560

[83] Chen S Wainwright DA Wu JD Wan Y Matei DE Zhang Y . CD73: an emerging checkpoint for cancer immunotherapy. Immunotherapy (2019) 11(11):983–97. doi: 10.2217/imt-2018-0200 PMC6609898 31223045

[84] Liu X Song J Zhang H Liu X Zuo F Zhao Y . Immune checkpoint HLA-E:CD94-NKG2A mediates evasion of circulating tumor cells from NK cell surveillance. Cancer Cell (2023) 41(2):272–87. doi: 10.1016/j.ccell.2023.01.001 36706761

[85] Xie J Gui X Deng M Chen H Chen Y Liu X . Blocking LAIR1 signaling in immune cells inhibits tumor development. Front Immunol (2022) 13:996026. doi: 10.3389/fimmu.2022.996026 36211388 PMC9534319

[86] Wight TN Kang I Evanko SP Harten IA Chang MY Pearce OMT . Versican-A critical extracellular matrix regulator of immunity and inflammation. Front Immunol (2020) 11:512. doi: 10.3389/fimmu.2020.00512 32265939 PMC7105702

[87] Zhang Q Wu J Chen X Zhao M Zhang D Gao F . Upregulation of versican associated with tumor progression, metastasis, and poor prognosis in bladder carcinoma. BioMed Res Int (2021) 2021:6949864. doi: 10.1155/2021/6949864 33604385 PMC7872746

[88] Hirani P Gauthier V Allen CE Wight TN Pearce OMT . Targeting versican as a potential immunotherapeutic strategy in the treatment of cancer. Front Oncol (2021) 11:712807. doi: 10.3389/fonc.2021.712807 34527586 PMC8435723

[89] Andreuzzi E Capuano A Poletto E Pivetta E Fejza A Favero A . Role of extracellular matrix in gastrointestinal cancer-associated angiogenesis. IJMS (2020) 21(10):3686. doi: 10.3390/ijms21103686 32456248 PMC7279269

[90] Lepucki A Orlińska K Mielczarek-Palacz A Kabut J Olczyk P Komosińska-Vassev K . The role of extracellular matrix proteins in breast cancer. J Clin Med (2022) 11(5):1250. doi: 10.3390/jcm11051250 35268340 PMC8911242

[91] Liot S Balas J Aubert A Prigent L Mercier-Gouy P Verrier B . Stroma involvement in pancreatic ductal adenocarcinoma: an overview focusing on extracellular matrix proteins. Front Immunol (2021) 12:612271. doi: 10.3389/fimmu.2021.612271 33889150 PMC8056076

[92] Tucker RP Degen M . Revisiting the tenascins: exploitable as cancer targets? Front Oncol (2022) 12:908247. doi: 10.3389/fonc.2022.908247 35785162 PMC9248440

[93] Yoshida KI Midwood KS Orend G . Editorial: tenascins – key players in tissue homeostasis and defense. Front Immunol (2022) 12:834353. doi: 10.3389/fimmu.2021.834353 35095934 PMC8790525

[94] Pyagay P Heroult M Wang Q Lehnert W Belden J Liaw L . Collagen triple helix repeat containing 1, a novel secreted protein in injured and diseased arteries, inhibits collagen expression and promotes cell migration. Circ Res (2005) 96(2):261–8. doi: 10.1161/01.RES.0000154262.07264.12 15618538

[95] Duan X Yuan X Yao B Song W Li Z Enhejirigala . The role of CTHRC1 in promotion of cutaneous wound healing. Signal Transduction Targeted Ther (2022) 7(1):183. doi: 10.1038/s41392-022-01008-9 PMC9197944 35701414

[96] Mei D Zhu Y Zhang L Wei W . The role of CTHRC1 in regulation of multiple signaling and tumor progression and metastasis. Mediators Inflamm (2020) 2020:9578701. doi: 10.1155/2020/9578701 32848510 PMC7441421

[97] Zhang XL Hu LP Yang Q Qin WT Wang X Xu CJ . CTHRC1 promotes liver metastasis by reshaping infiltrated macrophages through physical interactions with TGF-β receptors in colorectal cancer. Oncogene (2021) 40(23):3959–73. doi: 10.1038/s41388-021-01827-0 33986509

[98] Matsuda S Iriyama C Yokozaki S Ichigotani Y Shirafuji N Yamaki K . Cloning and sequencing of a novel human gene that encodes a putative target protein of Nesh-SH3. J Hum Genet (2001) 46(8):483–6. doi: 10.1007/s100380170049 11501947

[99] Delfín DA DeAguero JL McKown EN . The extracellular matrix protein ABI3BP in cardiovascular health and disease. Front Cardiovasc Med (2019) 6:23. doi: 10.3389/fcvm.2019.00023 30923710 PMC6426741

[100] Latini FRM Hemerly JP Freitas BCG Oler G Riggins GJ Cerutti JM . ABI3 ectopic expression reduces in *vitro* and in *vivo* cell growth properties while inducing senescence. BMC Cancer (2011) 11:11. doi: 10.1186/1471-2407-11-11 21223585 PMC3032749

[101] Lin N Yao Z Xu M Chen J Lu Y Yuan L . Long noncoding RNA MALAT1 potentiates growth and inhibits senescence by antagonizing ABI3BP in gallbladder cancer cells. J Exp Clin Cancer Res (2019) 38(1):244. doi: 10.1186/s13046-019-1237-5 31174563 PMC6555920

[102] Cai H Li Y Qin D Wang R Tang Z Lu T . The depletion of ABI3BP by microRNA-183 promotes the development of esophageal carcinoma. Mediators Inflamm (2020) 2020:3420946. doi: 10.1155/2020/3420946 32774142 PMC7399787

[103] Nong B Guo M Wang W Songyang Z Xiong Y . Comprehensive analysis of large-scale transcriptomes from multiple cancer types. Genes (Basel) (2021) 12(12):1865. doi: 10.3390/genes12121865 34946814 PMC8701385

[104] Ding Y Li P Wang W Liu F . A potential four-gene signature and nomogram for predicting the overall survival of papillary thyroid cancer. Dis Markers (2022) 2022:8735551. doi: 10.1155/2022/8735551 36193505 PMC9526076

[105] Bot S Andreuzzi E Capuano A Schiavinato A Colombatti A Doliana R . Multiple-interactions among EMILIN1 and EMILIN2 N- and C-terminal domains. Matrix Biol (2015) 41:44–55. doi: 10.1016/j.matbio.2014.10.001 25445627

[106] Capuano A Pivetta E Sartori G Bosisio G Favero A Cover E . Abrogation of EMILIN1-β1 integrin interaction promotes experimental colitis and colon carcinogenesis. Matrix Biol (2019) 83:97–115. doi: 10.1016/j.matbio.2019.08.006 31479698

[107] Pivetta E Capuano A Vescovo M Scanziani E Cappelleri A Rampioni Vinciguerra GL . EMILIN-1 deficiency promotes chronic inflammatory disease through TGFβ signaling alteration and impairment of the gC1q/α4β1 integrin interaction. Matrix Biol (2022) 111:133–52. doi: 10.1016/j.matbio.2022.06.005 35764213

[108] Marastoni S Andreuzzi E Paulitti A Colladel R Pellicani R Todaro F . EMILIN2 down-modulates the Wnt signalling pathway and suppresses breast cancer cell growth and migration. JPathol (2014) 232(0022-3417(0022-3417 (Linking):391–404. doi: 10.1002/path.4316 24374807

[109] Paulitti A Andreuzzi E Bizzotto D Pellicani R Tarticchio G Marastoni S . The ablation of the matricellular protein EMILIN2 causes defective vascularization due to impaired EGFR-dependent IL-8 production affecting tumor growth. Oncogene (2018) 37(25):3399–414. doi: 10.1038/s41388-017-0107-x 29483644

[110] Lorenzon E Colladel R Andreuzzi E Marastoni S Todaro F Schiappacassi M . MULTIMERIN2 impairs tumor angiogenesis and growth by interfering with VEGF-A/VEGFR2 pathway. Oncogene (2012) 31(26):3136–47. doi: 10.1038/onc.2011.487 22020326

[111] Andreuzzi E Capuano A Pellicani R Poletto E Doliana R Maiero S . Loss of multimerin-2 and EMILIN-2 expression in gastric cancer associate with altered angiogenesis. Int J Mol Sci (2018) 19(12):3983. doi: 10.3390/ijms19123983 30544909 PMC6321373

[112] Pellicani R Poletto E Andreuzzi E Paulitti A Doliana R Bizzotto D . Multimerin-2 maintains vascular stability and permeability. Matrix Biol (2020) 87:11–25. doi: 10.1016/j.matbio.2019.08.002 31422156

[113] Diehl V Huber LS Trebicka J Wygrecka M Iozzo RV Schaefer L . The role of decorin and biglycan signaling in tumorigenesis. Front Oncol (2021) 11:801801. doi: 10.3389/fonc.2021.801801 34917515 PMC8668865

[114] Sethi MK Downs M Shao C Hackett WE Phillips JJ Zaia J . In-depth matrisome and glycoproteomic analysis of human brain glioblastoma versus control tissue. Mol Cell Proteomics (2022) 21(4):100216. doi: 10.1016/j.mcpro.2022.100216 35202840 PMC8957055

[115] Liu M Wang W Piao S Shen Y Li Z Ding W . Relationship of biglycan and decorin expression with clinicopathological features and prognosis in patients with oral squamous cell carcinoma. J Oral Pathol Med (2023) 52(1):20–8. doi: 10.1111/jop.13381 36308714

[116] Zhao SF Yin XJ Zhao WJ Liu LC Wang ZP . Biglycan as a potential diagnostic and prognostic biomarker in multiple human cancers. Oncol Lett (2020) 19(3):1673–82. doi: 10.3892/ol.2020.11266 PMC7039163 32194659

[117] Hu S Li P Wang C Liu X . Expression and the prognostic value of biglycan in gastric cancer. Comput Math Methods Med (2022) 2022:2656480. doi: 10.1155/2022/2656480 36110576 PMC9470332

[118] Zhao L Liang J Zhong W Han C Liu D Chen X . Expression and prognostic analysis of BGN in head and neck squamous cell carcinoma. Gene (2022) 827:146461. doi: 10.1016/j.gene.2022.146461 35358652

[119] Bastos ACS da F Gomes AVP Silva GR Emerenciano M Ferreira LB Gimba ERP . The intracellular and secreted sides of osteopontin and their putative physiopathological roles. Int J Mol Sci (2023) 24(3):2942. doi: 10.3390/ijms24032942 36769264 PMC9917417

[120] Zhao H Chen Q Alam A Cui J Suen KC Soo AP . The role of osteopontin in the progression of solid organ tumour. Cell Death Dis (2018) 9(3):1–15. doi: 10.1038/s41419-018-0391-6 29500465 PMC5834520

[121] Zhang Y Du W Chen Z Xiang C . Upregulation of PD-L1 by SPP1 mediates macrophage polarization and facilitates immune escape in lung adenocarcinoma. Exp Cell Res (2017) 359(2):449–57. doi: 10.1016/j.yexcr.2017.08.028 28830685

[122] Klement JD Paschall AV Redd PS Ibrahim ML Lu C Yang D . An osteopontin/CD44 immune checkpoint controls CD8+ T cell activation and tumor immune evasion. J Clin Invest (2018) 128(12):5549–60. doi: 10.1172/JCI123360 PMC6264631 30395540

[123] Wei J Marisetty A Schrand B Gabrusiewicz K Hashimoto Y Ott M . Osteopontin mediates glioblastoma-associated macrophage infiltration and is a potential therapeutic target. J Clin Invest (2019) 129(1):137–49. doi: 10.1172/JCI121266 PMC6307970 30307407

[124] Spicer AP Joo A Bowling RA . A hyaluronan binding link protein gene family whose members are physically linked adjacent to chrondroitin sulfate proteoglycan core protein genes: THE MISSING LINKS *. J Biol Chem (2003) 278(23):21083–91. doi: 10.1074/jbc.M213100200 12663660

[125] Kuo SJ Chien SY Lin C Chan SE Tsai HT Chen DR . Significant elevation of CLDN16 and HAPLN3 gene expression in human breast cancer. Oncol Rep (2010) 24(3):759–66. doi: 10.3892/or_00000918 20664984

[126] Ding Y Xiong S Chen X Pan Q Fan J Guo J . HAPLN3 inhibits apoptosis and promotes EMT of clear cell renal cell carcinoma *via* ERK and Bcl-2 signal pathways. J Cancer Res Clin Oncol (2023) 149(1):79–90. doi: 10.1007/s00432-022-04421-3 36374334 PMC11796846

[127] Bjerre MT Nørgaard M Larsen OH Jensen SØ Strand SH Østergren P . Epigenetic analysis of circulating tumor DNA in localized and metastatic prostate cancer: evaluation of clinical biomarker potential. Cells (2020) 9(6):1362. doi: 10.3390/cells9061362 32486483 PMC7349912

[128] He Y Liu T Dai S Xu Z Wang L Luo F . Tumor-associated extracellular matrix: how to be a potential aide to anti-tumor immunotherapy? Front Cell Dev Biol (2021) 9:739161. doi: 10.3389/fcell.2021.739161 34733848 PMC8558531

[129] Nicolas-Boluda A Vaquero J Vimeux L Guilbert T Barrin S Kantari-Mimoun C . Tumor stiffening reversion through collagen crosslinking inhibition improves T cell migration and anti-PD-1 treatment. Elife (2021) 10. doi: 10.7554/eLife.58688.sa2 PMC8203293 34106045

[130] Kasashima H Yashiro M Okuno T Miki Y Kitayama K Masuda G . Significance of the lysyl oxidase members lysyl oxidase like 1, 3, and 4 in gastric cancer. Gastroenterologia (2018) 98(4):238–48. doi: 10.1159/000489558 30045039

[131] Tenti P Vannucci L . Lysyl oxidases: linking structures and immunity in the tumor microenvironment. Cancer Immunol Immunother (2020) 69(2):223–35. doi: 10.1007/s00262-019-02404-x PMC7000489 31650200

[132] Lu X Xin DE Du JK Zou QC Wu Q Zhang YS . Loss of LOXL2 promotes uterine hypertrophy and tumor progression by enhancing H3K36ac-dependent gene expression. Cancer Res (2022) 82(23):4400–13. doi: 10.1158/0008-5472.CAN-22-0848 36197797

[133] Xia QX Yu J Wang ZJ Guan QW Mao XY . Identification and validation of roles of lysyl oxidases in the predictions of prognosis, chemotherapy and immunotherapy in glioma. Front Pharmacol (2022) 13:990461. doi: 10.3389/fphar.2022.990461 36160460 PMC9490755

[134] Park KC Dharmasivam M Richardson DR . The role of extracellular proteases in tumor progression and the development of innovative metal ion chelators that inhibit their activity. Int J Mol Sci (2020) 21(18):6805. doi: 10.3390/ijms21186805 32948029 PMC7555822

[135] Lai YL Gong CL Fu CK Yueh TC Tsai CW Chang WS . The contribution of matrix metalloproteinase-1 genotypes to hepatocellular carcinoma susceptibility in Taiwan. Cancer Genomics Proteomics (2017) 14(2):119–25. doi: 10.21873/cgp.20024 PMC5369311 28387651

[136] Ueno M Shiomi T Mochizuki S Chijiiwa M Shimoda M Kanai Y . ADAM9 is over-expressed in human ovarian clear cell carcinomas and suppresses cisplatin-induced cell death. Cancer Sci (2018) 109(2):471–82. doi: 10.1111/cas.13469 PMC5797829 29247567

[137] Hubeau C Rocks N Cataldo D . ADAM28: Another ambivalent protease in cancer. Cancer Lett (2020) 494:18–26. doi: 10.1016/j.canlet.2020.08.031 32861707

[138] Ao T Mochizuki S Kajiwara Y Yonemura K Shiraishi T Nagata K . Cancer-associated fibroblasts at the unfavorable desmoplastic stroma promote colorectal cancer aggressiveness: Potential role of ADAM9. Int J Cancer (2022) 150(10):1706–21. doi: 10.1002/ijc.33947 35080810

[139] Jain R Gosavi S Sethia D Trimuke A Salunke M . Evaluation of expression of ADAM 10 as a predictor of lymph node metastasis in oral squamous cell carcinoma-an immunohistochemical study. Head Neck Pathol (2022) 16(4):1055–62. doi: 10.1007/s12105-022-01466-1 PMC9729510 35771404

[140] Kalita A Sikora-Skrabaka M Nowakowska-Zajdel E . Role of some microRNA/ADAM proteins axes in gastrointestinal cancers as a novel biomarkers and potential therapeutic targets-A review. Curr Issues Mol Biol (2023) 45(4):2917–36. doi: 10.3390/cimb45040191 PMC10136553 37185715

[141] Sharma D Singh NK . The biochemistry and physiology of A disintegrin and metalloproteinases (ADAMs and ADAM-TSs) in human pathologies. Rev Physiol Biochem Pharmacol (2023) 184:69–120. doi: 10.1007/112_2021_67 35061104

[142] Willumsen N Bager CL Leeming DJ Smith V Karsdal MA Dornan D . Extracellular matrix specific protein fingerprints measured in serum can separate pancreatic cancer patients from healthy controls. BMC Cancer (2013) 13:554. doi: 10.1186/1471-2407-13-554 24261855 PMC4222497

[143] Willumsen N Ali SM Leitzel K Drabick JJ Yee N Polimera HV . Collagen fragments quantified in serum as measures of desmoplasia associate with survival outcome in patients with advanced pancreatic cancer. Sci Rep (2019) 9(1):19761. doi: 10.1038/s41598-019-56268-3 31875000 PMC6930304

[144] Karsdal MA Nielsen SH Leeming DJ Langholm LL Nielsen MJ Manon-Jensen T . The good and the bad collagens of fibrosis - Their role in signaling and organ function. Adv Drug Delivery Rev (2017) 121:43–56. doi: 10.1016/j.addr.2017.07.014 28736303

[145] Nielsen MJ Nedergaard AF Sun S Veidal SS Larsen L Zheng Q . The neo-epitope specific PRO-C3 ELISA measures true formation of type III collagen associated with liver and muscle parameters. Am J Transl Res (2013) 5(3):303–15. PMC3633973 23634241

[146] Kehlet SN Sanz-Pamplona R Brix S Leeming DJ Karsdal MA Moreno V . Excessive collagen turnover products are released during colorectal cancer progression and elevated in serum from metastatic colorectal cancer patients. Sci Rep (2016) 6:30599. doi: 10.1038/srep30599 27465284 PMC4964349

[147] Lipton A Leitzel K Ali SM Polimera HV Nagabhairu V Marks E . High turnover of extracellular matrix reflected by specific protein fragments measured in serum is associated with poor outcomes in two metastatic breast cancer cohorts. Int J Cancer (2018) 143(11):3027–34. doi: 10.1002/ijc.31627 29923614

[148] Bager CL Willumsen N Leeming DJ Smith V Karsdal MA Dornan D . Collagen degradation products measured in serum can separate ovarian and breast cancer patients from healthy controls: A preliminary study. Cancer biomark (2015) 15(6):783–8. doi: 10.3233/CBM-150520 PMC12965475 26406420

[149] Hiebert PR Wu D Granville DJ . Granzyme B degrades extracellular matrix and contributes to delayed wound closure in apolipoprotein E knockout mice. Cell Death Differ (2013) 20(10):1404–14. doi: 10.1038/cdd.2013.96 PMC3770318 23912712

[150] Vassiliadis E Oliveira CP Alvares-da-Silva MR Zhang C Carrilho FJ Stefano JT . Circulating levels of citrullinated and MMP-degraded vimentin (VICM) in liver fibrosis related pathology. Am J Transl Res (2012) 4(4):403–14. PMC3493028 23145208

[151] Willumsen N Bager CL Leeming DJ Smith V Christiansen C Karsdal MA . Serum biomarkers reflecting specific tumor tissue remodeling processes are valuable diagnostic tools for lung cancer. Cancer Med (2014) 3(5):1136–45. doi: 10.1002/cam4.303 PMC4302665 25044252

[152] Mortensen JH Guo X De Los Reyes M Dziegiel MH Karsdal MA Bay-Jensen AC . The VICM biomarker is released from activated macrophages and inhibited by anti-GM-CSFRα-mAb treatment in rheumatoid arthritis patients. Clin Exp Rheumatol (2019) 37(1):73–80. 30418117

[153] Romano E Kusio-Kobialka M Foukas PG Baumgaertner P Meyer C Ballabeni P . Ipilimumab-dependent cell-mediated cytotoxicity of regulatory T cells ex vivo by nonclassical monocytes in melanoma patients. Proc Natl Acad Sci U S A (2015) 112(19):6140–5. doi: 10.1073/pnas.1417320112 PMC4434760 25918390

[154] Kim SE Yun S Doh J . Effects of extracellular adhesion molecules on immune cell mediated solid tumor cell killing. Front Immunol (2022) 13:1004171. doi: 10.3389/fimmu.2022.1004171 36389663 PMC9647090

[155] Kattner P Strobel H Khoshnevis N Grunert M Bartholomae S Pruss M . Compare and contrast: pediatric cancer versus adult Malignancies. Cancer Metastasis Rev (2019) 38(4):673–82. doi: 10.1007/s10555-019-09836-y 31832830

[156] Gupta A Cripe TP . Immunotherapies for pediatric solid tumors: A targeted update. Pediatr Drugs (2022) 24(1):1–12. doi: 10.1007/s40272-021-00482-y PMC8613512 34822115

[157] Carlino MS Larkin J Long GV . Immune checkpoint inhibitors in melanoma. Lancet (2021) 398(10304):1002–14. doi: 10.1016/S0140-6736(21)01206-X 34509219

[158] Huyghe N Baldin P Van den Eynde M . Immunotherapy with immune checkpoint inhibitors in colorectal cancer: what is the future beyond deficient mismatch-repair tumours? Gastroenterol Rep (Oxf) (2020) 8(1):11–24. doi: 10.1093/gastro/goz061 32104582 PMC7034232

[159] Farshbafnadi M Pastaki Khoshbin A Rezaei N . Immune checkpoint inhibitors for triple-negative breast cancer: From immunological mechanisms to clinical evidence. Int Immunopharmacol (2021) 98:107876. doi: 10.1016/j.intimp.2021.107876 34146865

[160] Izzi V Lakkala J Devarajan R Kääriäinen A Koivunen J Heljasvaara R . Pan-Cancer analysis of the expression and regulation of matrisome genes across 32 tumor types. Matrix Biol Plus (2019) 1:100004. doi: 10.1016/j.mbplus.2019.04.001 33543003 PMC7852311

[161] Edwards P Kang BW Chau I . Targeting the stroma in the management of pancreatic cancer. Front Oncol (2021) 11:691185. doi: 10.3389/fonc.2021.691185 34336679 PMC8316993

[162] Gordon-Weeks A Yuzhalin AE . Cancer extracellular matrix proteins regulate tumour immunity. Cancers (Basel) (2020) 12(11):3331. doi: 10.3390/cancers12113331 33187209 PMC7696558

[163] Rybchenko VS Aliev TK Panina AA Kirpichnikov MP Dolgikh DA . Targeted cytokine delivery for cancer treatment: engineering and biological effects. Pharmaceutics (2023) 15(2):336. doi: 10.3390/pharmaceutics15020336 36839658 PMC9960319

[164] Momin N Mehta NK Bennett NR Ma L Palmeri JR Chinn MM . Anchoring of intratumorally administered cytokines to collagen safely potentiates systemic cancer immunotherapy. Sci Transl Med (2019) 11(498). doi: 10.1126/scitranslmed.aaw2614 PMC7811803 31243150

[165] Lutz EA Jailkhani N Momin N Huang Y Sheen A Kang BH . Intratumoral nanobody–IL-2 fusions that bind the tumor extracellular matrix suppress solid tumor growth in mice. PNAS Nexus (2022) 1(5):pgac244. doi: 10.1093/pnasnexus/pgac244 36712341 PMC9802395

[166] Frey K Schliemann C Schwager K Giavazzi R Manfred J Neri D . The immunocytokine F8-IL2 improves the therapeutic performance of sunitinib in a mouse model of renal cell carcinoma. J Urol (2010) 184(6):2540–8. doi: 10.1016/j.juro.2010.07.030 21030045

[167] Lo KM Lan Y Lauder S Zhang J Brunkhorst B Qin G . huBC1-IL12, an immunocytokine which targets EDB-containing oncofetal fibronectin in tumors and tumor vasculature, shows potent anti-tumor activity in human tumor models. Cancer Immunol Immunother (2007) 56(4):447–57. doi: 10.1007/s00262-006-0203-1 PMC11030988 16874486

[168] Lei Y Li X Huang Q Zheng X Liu M . Progress and challenges of predictive biomarkers for immune checkpoint blockade. Front Oncol (2021) 11:617335. doi: 10.3389/fonc.2021.617335 33777757 PMC7992906

[169] Cui Y Zhang P Liang X Xu J Liu X Wu Y . Association of KDR mutation with better clinical outcomes in pan-cancer for immune checkpoint inhibitors. Am J Cancer Res (2022) 12(4):1766–83. PMC9077071 35530271

[170] Zhang P Pei S Liu J Zhang X Feng Y Gong Z . Cuproptosis-related lncRNA signatures: Predicting prognosis and evaluating the tumor immune microenvironment in lung adenocarcinoma. Front Oncol (2023) 12:1088931. doi: 10.3389/fonc.2022.1088931 36733364 PMC9887198

[171] Zhang P Pei S Gong Z Ren Q Xie J Liu H . The integrated single-cell analysis developed a lactate metabolism-driven signature to improve outcomes and immunotherapy in lung adenocarcinoma. Front Endocrinol (Lausanne) (2023) 14:1154410. doi: 10.3389/fendo.2023.1154410 37033259 PMC10073691

[172] Kovács SA Fekete JT Győrffy B . Predictive biomarkers of immunotherapy response with pharmacological applications in solid tumors. Acta Pharmacol Sin (2023) 44(9):1879–89. doi: 10.1038/s41401-023-01079-6 PMC10462766 37055532

[173] Ren Q Zhang P Zhang X Feng Y Li L Lin H . A fibroblast-associated signature predicts prognosis and immunotherapy in esophageal squamous cell cancer. Front Immunol (2023) 14:1199040. doi: 10.3389/fimmu.2023.1199040 37313409 PMC10258351

[174] Pei L Liu Y Liu L Gao S Gao X Feng Y . Roles of cancer-associated fibroblasts (CAFs) in anti- PD-1/PD-L1 immunotherapy for solid cancers. Mol Cancer (2023) 22(1):29. doi: 10.1186/s12943-023-01731-z 36759842 PMC9912573

